# The characterization of flavored hookahs aroma profile and in response to heating as analyzed *via* headspace solid-phase microextraction (SPME) and chemometrics

**DOI:** 10.1038/s41598-018-35368-6

**Published:** 2018-11-19

**Authors:** Mohamed A. Farag, Moamen M. Elmassry, Sherweit H. El-Ahmady

**Affiliations:** 1 Pharmacognosy Department, Faculty of Pharmacy, Cairo University, Cairo, Egypt; 2 Department of Chemistry, School of Sciences & Engineering, The American University in Cairo, New Cairo, 11835 Egypt; 3 Department of Biological Sciences, Texas Tech University, Lubbock, TX USA; 4 Pharmacognosy Department, Faculty of Pharmacy, Ain Shams University, Cairo, Egypt

## Abstract

Flavors profiling in flavored hookah tobacco is an issue of increasing scrutiny for the health sector owing to its adverse effects on humans, especially being heated to produce smoke. This study aims at tackling the components involved in the flavored hookah tobacco from a chemical and biological point of view. Detecting individual flavor compounds, within a complex hookah tobacco matrix was accomplished using headspace solid phase microextraction (SPME). A total of 114 volatiles were identified in 13 flavored hookah tobacco products, with esters amounting for the major component up to 40%. Whereas oxygenated monoterpenes presented another major volatile class, contributing up to 23%, including (*E*)-anethole. Superheating flavored hookah tobacco at 190 °C resulted in the release of a mixture of phenol derivatives and polycyclic aromatic compounds that are indicative of coal tar, a major component produced during hookah tobacco usage with potential health hazards. This study provides the first comprehensive volatile profile of hookah tobacco products from different origins identifying chemical components involved in flavors. It is expected to serve as informative grounds for the better understanding of hookah tobacco production and usage. The information presented is also expected to raise awareness on the health risks of hookah tobacco smoking.

## Introduction

Tobacco cigarette smoking is known for its adverse health effects including cancer, pulmonary and cardiovascular diseases^1^. Tobacco smoke comprises a large number of chemicals and brand-specific flavors, which contribute hundreds of volatiles, makes it a highly complex mixture. Many of these chemicals have been identified and characterized as toxins and/or carcinogens^2–4^. The increasing awareness of cigarette tobacco smoking health hazards has been shown to be one of the factors that led to a decrease in its consumption in 140 countries^5^. From 2000 to 2011, although per capita cigarette consumption decreased by 40.7%, non-cigarette combustible products consumption increased by 96.9%^6^. Among these non-cigarette combustible products is hookah (*aka*, waterpipe, shisha or narghile) tobacco, which its usage among the youth is on the rise^5–8^. In the United States, 18% of high school seniors reported hookah tobacco usage at least once a year^9^.

The mechanism of hookah tobacco smoking is unique. First, the tobacco is heated indirectly with charcoal, then the smoke passes through a water bowl and finally is inhaled by the smoker through a rubber hose fitted with a mouthpiece^7^. Hookah tobacco products come in different flavors, such as apple, mint, cherry, chocolate, coconut, licorice, cappuccino, and watermelon. Despite that hookah tobacco usage shares the same safety concerns as cigarettes, a misconception exists that it is safer and less addictive^9,10^. One of the major reasons for the popularity of hookah tobacco is its presentation in various pleasant flavors with distinct attractive aromas^11^. Food flavor additives are regulated and monitored by the U.S. Food and Drug Administration (FDA) for any possible adverse effects on humans. In the same context, hookah tobacco flavors profiling is vital for its quality control and safety measures. Recently, the FDA issued a notice of proposed rulemaking for regulating e-cigarettes, cigars, waterpipe tobacco, dissolvable tobacco, nicotine gels and all other products made or derived from tobacco^12^. Previous studies have focused on studying the smoke exposure through hookah tobacco in comparison with cigarettes, and identifying the chemical constituents of hookah tobacco, but very limited research has been conducted on flavored hookah tobacco products and their chemical analyses^13–16^. The fact that these flavors are appealing to adolescents worldwide and are regarded as “safe” seem to be the most conceivable reason for hookah tobacco consumption, and poses a very crucial target for investigation^17^.

**Figure 1:**
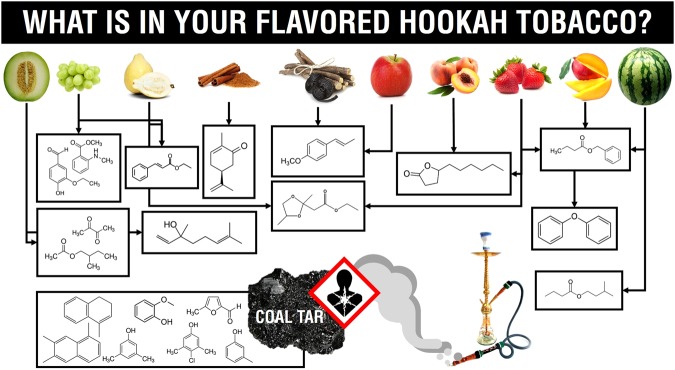
What is in your flavored hookah tobacco?

In this study, we aim to provide answers for the underlying question: What is in your flavored hookah tobacco? (Fig. 1). We employed the technique of headspace solid phase microextraction (SPME) coupled to gas chromatography-mass spectrometry (GC-MS) for volatiles profiling in 11 different flavored hookah tobacco products namely, apple, green grape, guava, melon, watermelon, strawberry, cinnamon, mango, peach, and unflavored “Kas” from different manufacturers, in addition to tobacco cigarette. These flavors were selected based on their wide availability and popularity as recommended by suppliers. To our knowledge, this study presents the first attempt to characterize aroma of the various commercial hookah tobacco flavors. For different flavors classification, multivariate data analyses were applied for the identification of unique chemicals contributing to the flavor of each hookah tobacco type^18–20^. Considering that hookah tobacco when consumed is heated with charcoal at temperature does not exceed 200 °C^21^, volatiles were collected from 5 different flavored hookah tobacco heated with charcoal and placed at 190 °C prior to the volatiles collection step. Such elevated temperature would allow for the assessment of the less volatile components resulting from burning^22^ in addition to the volatiles released from coal tar, a common by-product in hookah tobacco usage and regarded as a major health hazard^23,24^.

## Results

### GC-MS peaks identification in hookah tobacco products

**Figure 2:**
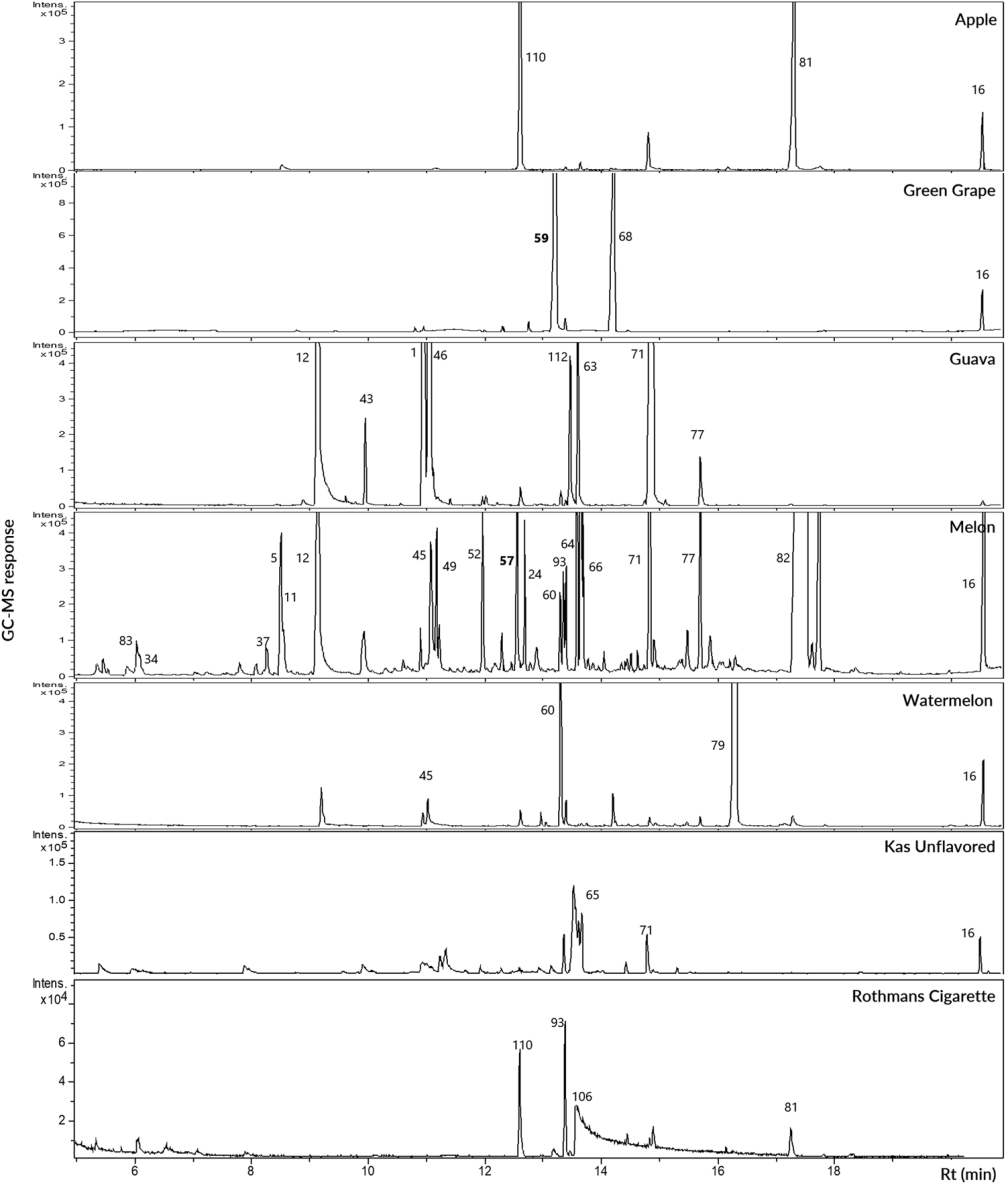
SPME-GC-MS representative chromatogram of headspace volatiles collected from apple, green grape, guava, melon, watermelon, unflavored “Kas” hookah Egyptian (EG) specimens and cigarette after brought hot with charcoal at 50 °C for 10 minutes. The corresponding compound names for volatile peaks follow that listed in Supplementary Table S1. 1, Unknown acetal; 5, Caproic acid; 11, 2-Ethyl-1-hexanol; 12, Benzyl alcohol; 13, Unknown alcohol; 16, Tetradecamethylene glycol; 24, Cinnamaldehyde; 34, 2-Methylbutyl acetate; 37, Ethyl caproate; 43, Amyl valerate; 46, Ethylacetoacetate propyleneglycol ketal; 49, *(E)*-2-Hexenyl Butyrate; 52, Linalyl acetate; 57, *(Z)*-6-Nonenyl acetate; 59, Triacetin; 60, Benzyl butanoate; 63, *(Z)*-β-Hexenyl Caproate; 64, Hexyl caproate; 65, Cinnamyl butyrate isomer; 66, *(E)*-2-Hexenyl caproate; 68, Methyl methanthranilate; 71, Ethyl cinnamate; 77, Benzyl hexanoate; 79, Cinnamyl isobutyrate; 81, Hedione; 82, α-Amylcinnamaldehyde; 83, 2,3-Butanedione; 93, (±)-Solanone; 106, Nicotine; 110, Anethole; 112, Eugenol. Rt, Retention time.

**Figure 3:**
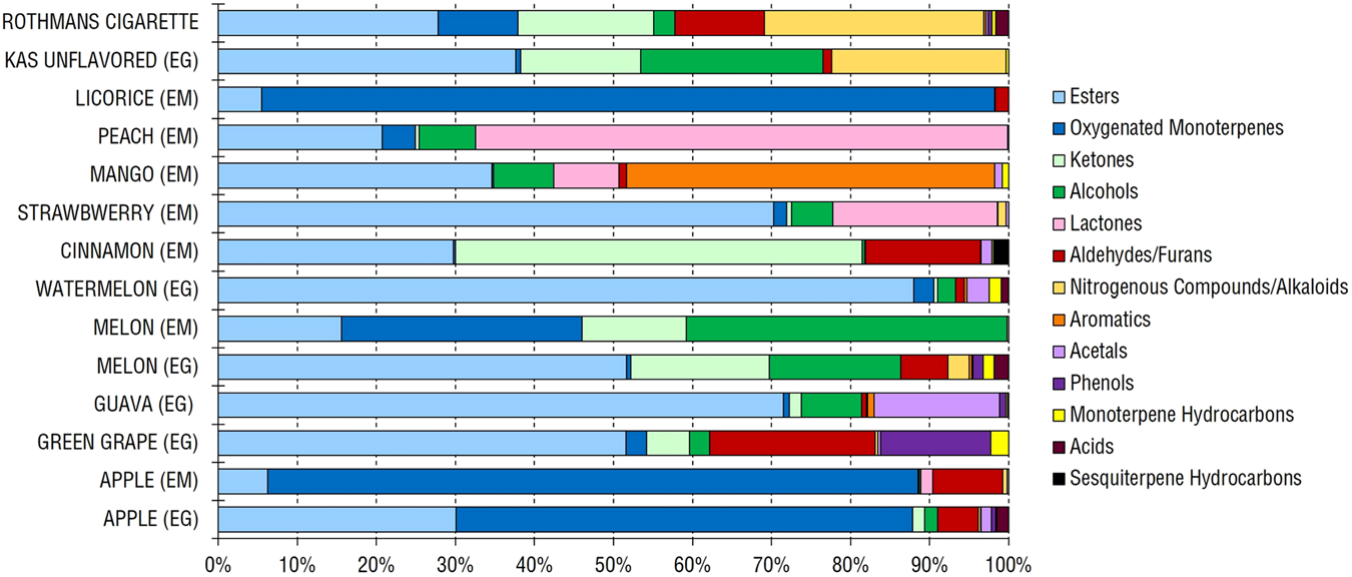
Major volatile classes in different unflavored and flavored hookah tobacco products and Rothmans cigarette. (EG) Egyptian hookah tobacco, (EM) Emirates hookah tobacco.

Volatiles collection using SPME led to the identification of 114 volatile components (Supplementary Table S1) belonging to 13 major classes of volatile compounds comprising esters, oxygenated monoterpenes, ketones, alcohols, lactones, aldehydes/furans, nitrogenous compounds/alkaloids, aromatics, acetals, phenols, monoterpene hydrocarbons, acids, and sesquiterpene hydrocarbons (Figs 2 and 3).

### Esters

Esters were the most abundant class of volatiles found in all flavored hookah tobacco with an average of 38.65% of the total volatile blend, except for the apple (EG and EM), licorice (EM), melon (EM), mango (EM), and peach (EM) flavors in which esters were the second most abundant class. Esters are commonly incorporated in flavored hookah and cigarette tobacco products to improve their odor^25,26^. Ethylacetoacetate propyleneglycol ketal was detected at high levels in guava (EG) flavored hookah tobacco at 42%, followed by lower levels in strawberry (EM), melon (EG) and watermelon (EG) flavored hookah tobacco. Ethylacetoacetate propyleneglycol ketal is a water contaminant that can readily form in aqueous solutions from the reaction of propanal and propylene glycol^27,28^ and whether it is formed as an artefact during volatiles humidification step during hookah aspiration has yet to be determined. Benzyl butanoate was mainly present in strawberry (EM) and watermelon (EG) flavored hookah tobacco at 14% followed by 11% in mango (EM) and 8% in apple (EG) flavored hookah tobacco. Benzyl butanoate was previously reported in electronic cigarette fluids^29,30^. 2-Methylbutyl acetate was detected in Kas unflavored hookah tobacco at 14%, and melon (EG and EM) flavored ones at 13% and 8%, respectively, and at slightly lower levels in Rothmans tobacco cigarette at 6%; it has an ethereal rum-like fermented-fruity odor found in electronic cigarette fluids^25,31^. iso-Amyl iso-butyrate was present at a high level only in watermelon (EG) flavored hookah tobacco at 35%. Ethyl cinnamate was rich in guava (EG) and green grape (EG) flavored hookah tobacco at 19% and 11, respectively. Ethyl cinnamate exhibits a fruity balsamic honey-like odor and is known to be the main aroma compounds in Pinot noir wine^32–34^. An ester predominating the Kas unflavored hookah tobacco was ethyl α-methylbutyrate at 22%. Ethyl α-methylbutyrate was previously reported in other flavored waterpipe tobaccos and wine^13,34^. Methyl methanthranilate, exhibiting a sweet fruity odor^33^, was found almost exclusively in green grape (EG) flavored hookah tobacco at 27%. 2-Methylbutyl butanoate constituted around 19% and 8% in watermelon (EG) and strawberry (EM) flavored hookah tobacco products, respectively. Ethyl phenylacetate, naturally occurring in many fruits such as apple, grapefruit and guava was found in apple (EG) and cinnamon (EM) flavored hookah tobacco at 13% and 7%, respectively^34^. *(Z)* and *(E)*-2-hexenyl butyrate was found exclusively in strawberry (EM) flavored hookah tobacco at 20% and 9%, respectively, while α,α-dimethylphenethyl butyrate was found exclusively in peach (EM) flavored hookah tobacco at 17%. Linalyl acetate in cinnamon (EM) flavored hookah tobacco at 16%. Benzyl n-heptanoate had a unique presence only in mango (EM) flavored hookah tobacco at 11%. α-Amylcinnamaldehyde was detected in melon (EG) and licorice (EM) flavored hookah tobacco at 5%, each. It is known for its pleasant cinnamon fragrance, however its inhalation may cause drowsiness and dizziness (TOXNET). Hedione (methyl dihydrojasmonate), a constituent in perfumery that act as a human pheromone leading to sex-differentiated hypothalamic activation and has potential aphrodisiac properties^35,36^ was detected in Rothmans tobacco cigarette at 5%. Cinnamyl isobutyrate was found exclusively in watermelon (EG) flavored hookah tobacco at 9% and is routinely detected as a fragrance in cosmetic products for its characteristic fruity, slightly floral odor^37^. Benzyl hexanoate was present significantly at 8% only in mango (EM) flavored hookah tobacco.

### Oxygenated monoterpenes

Oxygenated monoterpenes were the most abundant volatile class in apple (EG and EM) and licorice (EM) flavored hookah tobacco at 58%, 82% and 93%, respectively. On the other hand, this class contributed up to 30% to the volatiles composition of melon (EM) flavored hookah tobacco, followed by 10% in Rothmans tobacco cigarette. (*E*)-Anethole was the sole volatile constituent, of oxygenated monoterpenes, in apple flavored hookah tobacco found at high levels in EG (58%) and EM (82%), well-recognized as a marker flavor for apple flavored hookah tobacco. It was also the only predominant constituent of licorice (EM) flavored hookah tobacco at 93%. *(E)*-Anethole is sweet and is commonly found in alcoholic beverages and oral hygiene products such as gargles. We observed diepoxy-p-menthane in melon (EM) flavored hookah tobacco at 30%. Isomenthone, another oxygenated monoterpene, was only detected in green grape (EG) flavored hookah tobacco aroma profile at low level of 2%, which is used as a fragrance in many cosmetic products with wounding healing effect and was found to have a protective effect on fibroblast, cells of connective tissue, from tumor necrosis factor (TNF)-α-induced death^38^.

### Ketones

Ketones were found at 51% in cinnamon (EM) flavored hookah tobacco, mainly in its carvone content at 48%, which interestingly was non-existent in the rest of hookah tobacco products. Carvone, a natural volatile in caraway oil, is used in fragrance and flavor industries, with insecticidal effect^39,40^. 2,3-Butanedione was found in melon (EG and EM) flavored, Kas unflavored hookah tobacco, and Rothmans cigarette at 13%, 11%, 14%, and 6%, respectively. 2,3-Butanedione is well-known for its butter, caramel odor, naturally occurring as a fermentation by product, albeit can lead to several respiratory ailments such as, bronchiolitis obliterans, *aka* popcorn lung disease^41^. (±)-Solanone, a natural ketone found in tobacco leaves, was detected in Rothmans tobacco cigarette at 7% and is known to enhance its fragrance^42^.

### Alcohols

Alcohols are commonly identified as major aroma components in flavored hookah tobacco and cigarettes to improve their smoke smell^25,26^. Among alcohols, β-linalool was detected in melon (EM) flavored hookah tobacco at 32%. β-Linalool is often included as a fragrance in hygienic products owing to its fresh, flowery and citrus-like odor in addition to its insecticidal effect^39^ with a relatively low odor threshold of 0.0045 mg/L which indicates its significant contribution to the flavor^43^. Although β-linalool itself is non-irritant, it is susceptible to autoxidation and the oxidized β-linalool is allergenic^44^. Testing β-linalool on human cancer cell lines showed that it has a comparable cytotoxic effect to the commercial anticancer drug vinblastine^45^. *(E)*-2-hexenol was detected in a considerable level in Kas unflavored hookah tobacco at 22%, which is one of the green leaf volatiles emitted by plants after stress and exhibit a green type odor^25,46,47^. Benzyl alcohol is an allergenic fragrance used in cosmetics that was identified in mango (EM) flavored hookah tobacco around 8% and at lower levels in guava (EG), melon (EG), and strawberry (EM) flavored hookah tobacco products^13,48^. 1-Hexanol was found at a considerable level in melon (EG and EM) flavored hookah tobacco around 5% and 8%, respectively. Overexposure to this alcohol can lead to eye and respiratory tract irritation and central nervous system depression^49,50^.

### Lactones

Lactones were the most abundant class in peach (EM) flavored hookah tobacco at 67%. Two main lactones were present, γ-decalactone and γ-undecalactone. The former lactone with strong peach aroma, was found in a considerable level 51% in peach (EM) flavored hookah tobacco and it is used for flavoring beverages and food. While being present in lower levels in strawberry (EM) and mango (EM) flavored hookah tobacco at 21% and 8%, respectively. γ-Undecalactone was found only peach (EM) flavored hookah tobacco at a considerable level, 16%. γ-Decalactone and γ-undecalactone, naturally present in strawberries, were previously detected in strawberry-flavored tobacco products^2^.

### Aldehydes/Furans

Aldehydes are commonly identified in flavored hookah tobacco and cigarettes to enhance smoke smell^25^, and was found at highest levels in green grape flavored hookah tobacco at 21%. Ethyl vanillin is commonly used as an artificial vanilla flavoring agent to improve products sensory traits^51^, and was detected chiefly only in green grape (EG) flavored hookah tobacco (19%). *(E)*-Cinnamaldehyde, naturally occurring in cinnamon, was found in cinnamon (EM) flavored hookah tobacco at 14%. Owing to its characteristic taste and antimicrobial activity, it is commonly added in cosmetics^43^ and has additionally high safety margin (TOXNET). (*E*)-Cinnamaldehyde was also found in lower levels in apple (EG and EM) and melon (EG) at 4%, 9% and 5%, respectively. Several furans namely, furfural, 3-furaldehyde and 5-methyl furfural were detected at low levels 4%, 2% and 2%, respectively, in Rothmans cigarette. These furans are usually generated from sugars degradation and can cause pulmonary irritation upon inhalation^52–54^. Furans are commonly found in flavored tobacco cigarettes, such as cherry, grape and apple flavorings^4^ known to act as central nervous system depressants^53^.

### Nitrogenous compounds/Alkaloids

Nitrogenous compounds i.e., alkaloids were only abundant in Rothmans cigarette volatile blend and Kas unflavored hookah tobacco at 28% and 22%, respectively, although with difference in their volatile composition. Nicotine was the major volatile detected in Rothmans cigarette at 28% versus pyrrolidine abundance in Kas unflavored hookah tobacco at 22%. Nicotine is the known addictive substance in cigarette smoke^46^ and its toxicity is well-reported in many animal models including nausea, vomiting and paralysis at high doses, in addition to increasing incidence of lung cancer. Pyrrolidine is considered a health hazard (TOXNET)^55^ as upon inhalation it causes excitement and convulsions in mice. Even though nicotine was shown to support tobacco dependence in hookah users^56^, labelling it as a health hazard in Kas unflavored hookah tobacco has yet to be determined by analyzing specimens from other manufacturers and/or assessment of its toxic effects in animals. In terms of pyrrolidine flavor imparting effect, it has ammonia-like odor and was reported to be produced during brewing process^57^. Nicotine and pyrrolidine were also detected in a very low abundance in other flavored hookah tobacco products. Another toxic nitrogenous compound detected however at trace levels is nitrosoazetidine in Rothmans tobacco cigarette, apple (EG), and melon (EG) flavored hookah tobacco products. Nitrosoazetidine is categorized as a liver carcinogen in animals and its safety through inhalation needs to be further studied^58^.

### Aromatics

Aromatics, represented in diphenyl ether, were uniquely abundant in mango flavored hookah tobacco at around 47%. Diphenyl ether, which has a harsh metallic aroma, is irritant to the mucus membranes and the upper respiratory tract, and its prolonged exposure results in damage to multiple organs^59^.

### Phenols

Phenols were found at appreciable amounts (14%) in green grape (EG) flavored hookah tobacco, with eugenol as the major volatile component. Eugenol is a natural flavor found in plants *i*.*e*., clove with a spicy flavor, and its presence is considered beneficial in hookah by relieving smoking irritation and acting as a local anaesthetic^50^. Phenol, a health hazardous agent due to its high reactivity^52^ was detected at low levels (<1.5%) in Rothmans cigarette and apple (EG), green grape (EG), guava (EG), and melon (EG) flavored hookah tobacco products.

### Monoterpene hydrocarbons

Monoterpene hydrocarbons are a class of terpenes that are highly represented in plant essential oils. Limonene was detected at low level ≤1% in most flavored hookah tobacco products. It has a citrus-like odor, which accounts for its incorporation in cosmetic and food products^60^.

### Acids

Caproic acid was the main volatile acid detected at levels <2% in Rothmans cigarette, apple (EG), melon (EG) and watermelon (EG) flavored hookah tobacco, whereas benzoic acid at <1% in melon (EG) watermelon (EG), and guava (EG) flavored hookah tobacco. Caproic acid is a saturated medium-chain fatty acid of a cheese-like smell, naturally occurring in various plants and animal fats^61^. In contrast, benzoic acid has a balsamic odor in addition to its common use as a food preservative. If inhaled as in case of hookah smoke, it can cause respiratory tract irritation^30^, and whether their presence in hookah tobacco aroma is a health hazard has yet to be critically assessed.

### Multivariate data analysis of flavored hookah tobacco volatiles data

**Figure 4:**
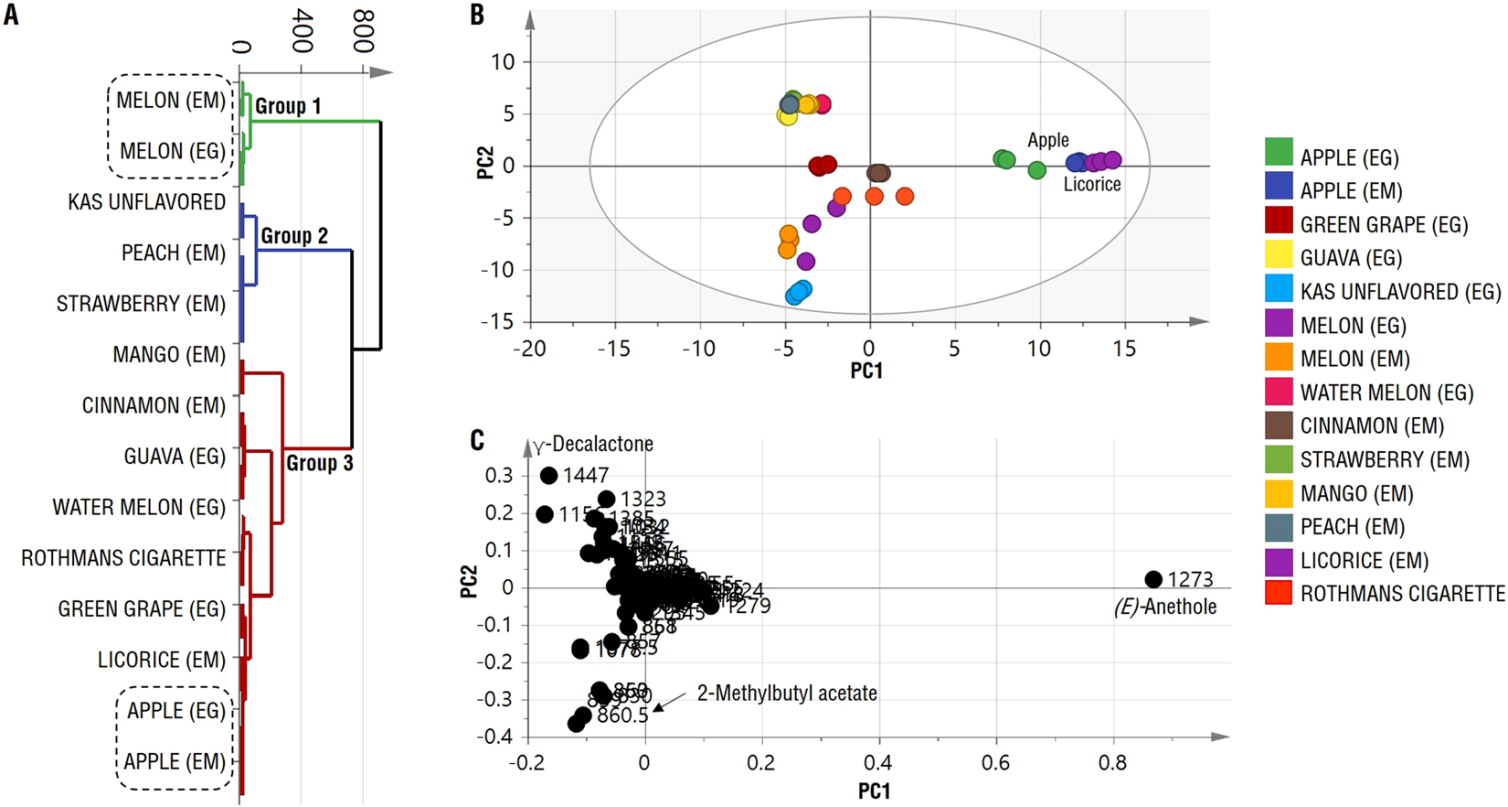
Hierarchical clustering analysis (HCA) and principal component analysis (PCA) analyses of SPME extracted volatile. (**A**) HCA plot, (**B**) PCA score plot of PC1 vs. PC2 scores and (**C**) Loading plot for PC1 & PC2 contributing volatiles and their assignments. (EG) Egyptian hookah tobacco, (EM) Emirates hookah tobacco.

Owing to the complexity of the acquired data encompassing both the number of flavor sample size (14 specimens, each represented by 3 replicates) (Supplementary Fig. S1) and monitored volatiles, multivariate data analyzes were performed on flavored hookah tobacco volatile profiles to better define similarities and differences among them in an untargeted manner and to ensure good analytical rigorousness. In our case, this will serve to find unique volatiles or markers as well as pinpoint known potential hazardous volatiles in each flavored product. Hierarchical clustering analysis (HCA) grouped hookah flavors in three major clusters (Fig. 4A), the first includes melon samples, the second comprises strawberry, peach and Kas unflavored samples, and the third includes the rest. The HCA plot also showed the clustering of hookah tobacco specimens from different origins, as in the case of the apple and melon flavored hookah tobacco products obtained from Egypt (EG) and United Arab Emirates (EM) which confirms the similarity of the products regardless of their origins.

Examination of the PCA score plot (Fig. 4B) prescribed by PC1 and PC2, revealed that apple and licorice samples clustered closely and distinctly from other samples. In terms of hookah specimens clustering close to Rothmans cigarette; cinnamon (EM), green grape (EG), and melon (EG and EM) were the closest. The loading plot (Fig. 4C), which represents the most important volatile components with respect to scattering pattern, revealed that *(E)*-anethole enrichment in licorice and apple flavors accounted for its role in segregating different samples along PC1. However, *(E)*-anethole shifted the distribution of the other volatiles through the loading plot, thus were-generated the PCA and loading plot after removal of *(E)*-anethole peak from the dataset to have a clearer picture of other less abundant volatiles distribution among hookah specimens.

**Figure 5:**
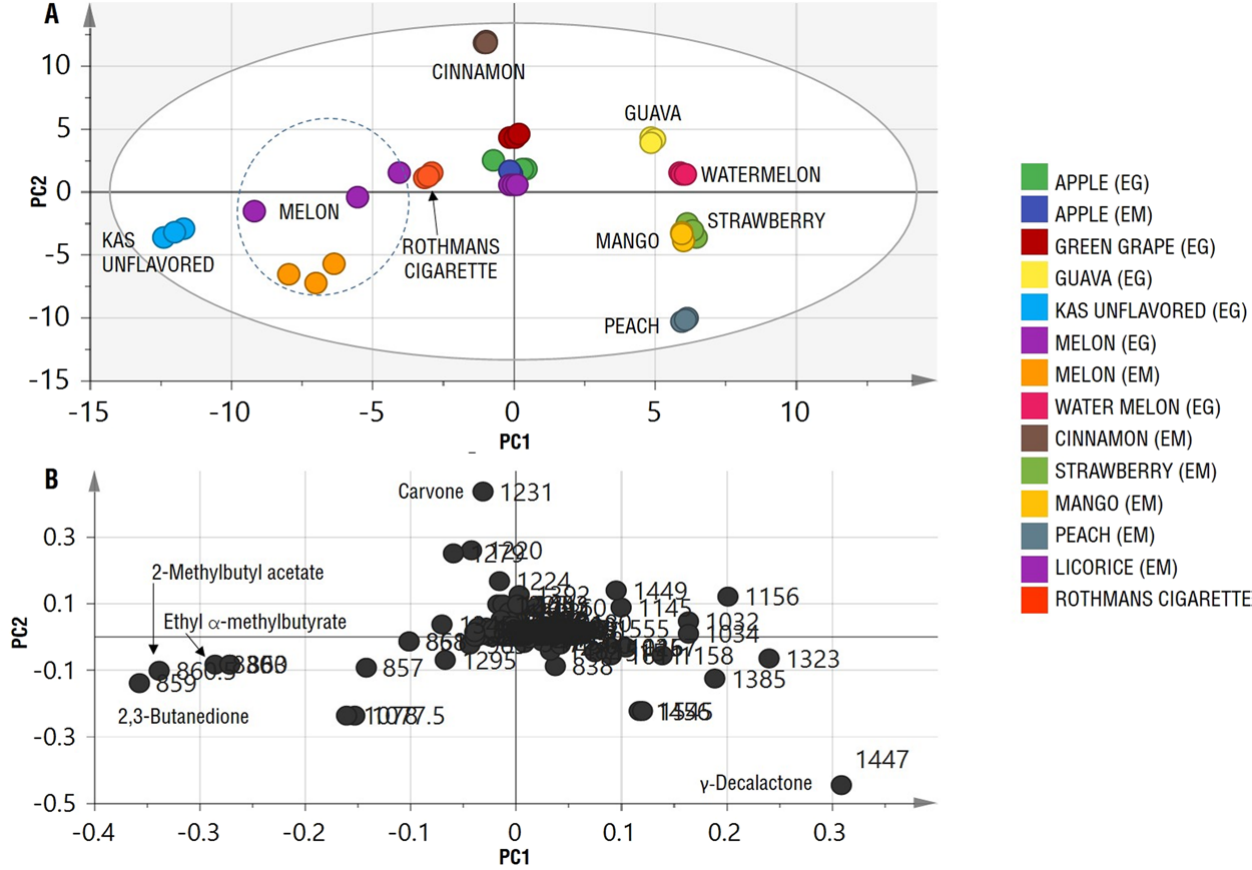
Principal component analysis (PCA) analyses of SPME extracted volatile collected on cold after exclusion of (*E*)-anethole peak abundance from data matrix. (**A**) PCA score plot of PC1 vs. PC2 scores. and (**B**) Loading plot for PC1 & PC2 contributing volatiles and their assignments. (EG) Egyptian flavours. (EM) Emirates flavours.

Examination of the new PCA score plot (Fig. 5A), led to clearer separation of the specimens. Along PC2 cinnamon and peach samples were clustered separately due to their high levels of carvone and γ-decalactone, respectively, which is revealed from the loading plot (Fig. 5B). Also, the melon samples were the closest to the Rothmans cigarette samples, as well as the Kas unflavored samples (Fig. 5A). Moreover, the separation of the Kas unflavored samples along PC1 is attributed their considerable content in 2,3-butanedione, ethyl α-methylbutyrate, and 2-methylbutyl acetate (Fig. 5B).

### Multivariate data analysis of cigarette smoke compared to hookah tobacco products

**Figure 6:**
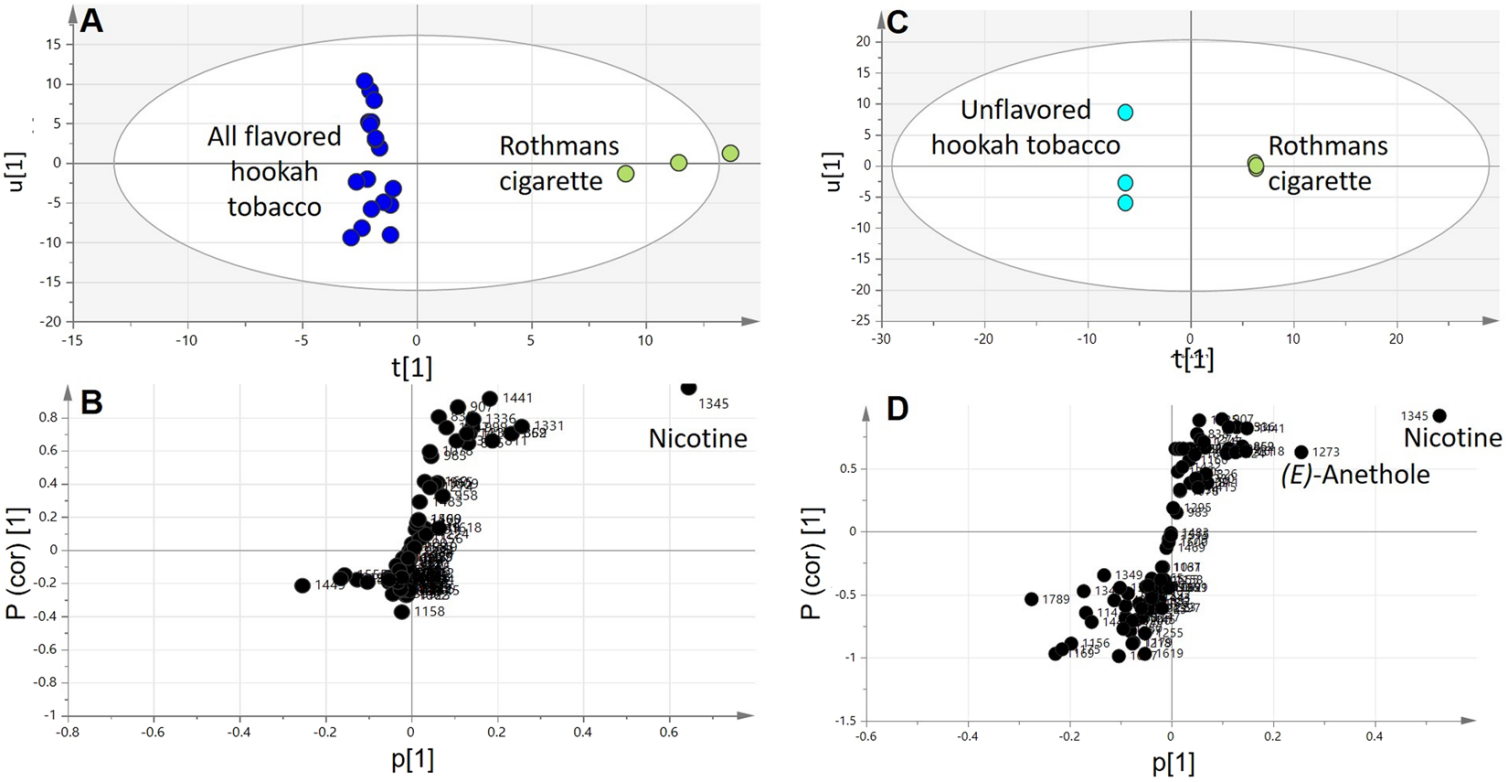
OPLS-DA score plot derived from modelling cigarette aroma versus hookah flavours (**A**) and cigarette aroma versus unflavoured “Kas” brand (**C**) each modelled one at a time. The respective S-plot (**B**,**D**) shows the covariance p[1] against the correlation p(cor)[1] of the variables of the discriminating component of the OPLS-DA model. Cut-off values of P < 0.05 were used; selected variables are highlighted in the *S*-plot with kovats index value and identifications are discussed in text.

To better assess the difference between cigarette volatiles profile from that of unflavored and flavored hookah tobacco which we could not disentangle from PCA, supervised orthogonal projection to latent structures-discriminant analysis (OPLS-DA) was used to build a classification model for distinguishing between hookah flavor and cigarette. OPLS-DA also has greater potential in the identification of markers by providing the most relevant variables for the differentiation between two sample groups. Cigarette was modelled against all other hookah flavors modelled against as one class group, with the derived score plot showing a clear separation between both samples (Fig. 6). The OPLS score plot explained 83% of the total variance (R^2^ = 0.83) with the prediction goodness parameter Q^2^ = 0.75 (Fig. 6). A particularly useful tool in OPLS-DA that provides variable importance in projection (VIP) scores for each volatile and describes a quantitative measure of the discriminatory power of each contributing component flavor. The OPLS-DA model was validated using the diagnostic metrics Q2, R2, permutation testing, and *P*-value to avoid overfitting and assess the statistical significance of the model (Supplementary Fig. 1)^20^. The selected features (volatiles) had the highest VIP scores^19,62^. Top 10 major compounds identified with VIP scores >1.5 (Supplementary Spreadsheet S1) as revealed from OPLS-DA that are characteristic to tobacco cigarette and have the potential to discriminate it from hookah tobacco products include: Furfuryl alcohol, (±)-solanone, dihydrocarvyl acetate, 5-methylfurfural, nicotine, furfural, nitrosoazetidine, 3-furaldehyde, *(Z)*-3-hexenyl heptanoate, geranyl acetone, hedione (methyl dihydrojasmonate), piperonal, *(Z)*-β-hexenyl caproate, γ-ionone, and geraniol butyrate. It is worth noting that 5 of these aforementioned components are reported to bear irritant characteristic and hazards to health as mentioned previously.

### Multivariate data analysis of hookah tobacco products and their top predictive volatiles

To further reveal whether each hookah volatile fingerprint was unique enough to be identified as markers for each flavor, OPLS-DA modelling was applied. Each flavor was modelled separately; one at a time against all other samples present in one class group to identify the top volatiles correlated with each hookah flavor and had high predictive ability. The OPLS-DA models were validated, as previously described (Supplementary Fig. S2). From these models, only volatiles with VIP score ≥1.5 (Supplementary Spreadsheet S1) were annotated to ensure their predictive validity. Listed below are the key volatiles for each hookah tobacco product that have high predictive value of the flavor associated with reference to any possible health hazards reported.

#### Kas unflavored hookah tobacco

*(E)*-2-Hexenol, pyrrolidine (health hazard)^35,58^, ethyl caprate, 2-methylbutyl acetate, and 2,3-butanedione (health hazard)^44^.

#### Apple (EG) flavored hookah tobacco

Benzyl propionate, isomenthol acetate, 2-heptyl-1,3-dioxane, ethyl vanillin, 2-hexenol acetate, caproic acid (health hazard)^31^, and *(E)*-anethole.

#### Apple (EM) flavored hookah tobacco

Hedione (methyl dihydrojasmonate), nicotine (health hazard)^56^, *(E)*-anethole, trans-2-hexenyl caproate, cinnamaldehyde, and *(Z)*-6-nonenyl acetate.

#### Green grape (EG) flavored hookah tobacco

5-Acetoxymethyl-2-furaldehyde, 2-hydroxy-3-isopropyl-6-methyl-2-cyclohexen-1-one, diosphenol, methyl methanthranilate, propyl isobutyrate, isomenthone, triacetin, *(E)*-β-ocimene, ethyl α-methylbutyrate, eugenol, methyl salicylate, piperonal, α-phenylethyl acetate, and *(Z)*-β-ocimene and hexyl caproate.

#### Guava (EG) flavored hookah tobacco

Pyranone, 1-phenylbutadiene, ethylacetoacetate propyleneglycol ketal, isobutyl caproate, dihydrocarvyl acetate, 4,7-dimethylbenzofuran, *(Z)*-β-hexenyl caproate, ethyl cinnamate, α-terpineol, hydrocinnamyl isobutyrate, and geraniol butyrate.

#### Melon (EG) flavored hookah tobacco

n-Hexyl acetate, n-butyl butyrate, 5-methyl furfural (health hazard)^39–41^, ethyl phenylacetate, butyl caproate, nitrosoazetidine (health hazard)^58^, benzyl acetate, benzoic acid (health hazard)^31^, 2-ethyl-1-hexanol, isoamyl caproate, benzyl formate, amyl valerate, β-myrcene, isoterpinolene, phenol (health hazard)^55^, butenone, iso-amyl iso-butyrate, *(Z)*-6-nonenyl acetate, *(E)*-2-hexenyl caproate, 2-hexenol acetate, and β-acetonaphthone.

#### Melon (EM) flavored hookah tobacco

Propyl methacrylate, diepoxy-p-menthane, β-linalool (health hazard)^44^, 3-hexen-1-ol, 6-methyl-5-hepten-2-one, and 2,3-butanedione (health hazard)^44^.

#### Watermelon (EG) flavored hookah tobacco

2-Furfuryl-5-methylfuran (health hazard)^31,39,40,52^, isoamyl acetate, cinnamyl isobutyrate, ethyl caproate, 2-methylbutyl butyrate, benzyl butanoate, *(Z)*-β-ocimene, limonene, menthol, α-phenylethyl acetate, and vanillin.

#### Cinnamon (EM) flavored hookah tobacco

(*−*)-β-Bourbonene, caryophyllene, carvone, linalyl acetate, tetradecamethylene glycol, pulegone, geranyl acetone, menthyl acetate, menthol, cinnamaldehyde, (±)-solanone, cineole, γ-ionone, and geraniol butyrate.

#### Strawberry (EM) flavored hookah tobacco

*(Z)*-3-Hexenyl butyrate, *(E)*-2-hexenyl butyrate, cinnamyl butyrate isomer, furaneol, furfural (health hazard)^39,40,52^, benzyl butanoate, 3-furaldehyde (health hazard)^39,40,52^.

#### Mango (EM) flavored hookah tobacco

Benzyl hexanoate, benzyl n-heptanoate, cinnamyl butyrate, diphenyl ether (health hazard)^59^, citronellyl butyrate, benzyl alcohol (health hazard)^48^, benzaldehyde, benzaldehyde propylene glycol acetal, and geranyl isobutyrate.

#### Peach (EM) flavored hookah tobacco

γ-Undecalactone, α,α-dimethylphenethyl butyrate, γ-decalactone, 1-hexanol (health hazard)^49,50^, *(Z)*-3-hexenyl heptanoate, and citronellyl butyrate.

#### Licorice (EM) flavored hookah tobacco

p-Anisaldehyde, *(E)-*anethole, α-amylcinnamaldehyde (health hazard) (TOXNET), and *(Z)*-6-nonenyl acetate.

### Multivariate data analysis of flavored hookah tobacco heated up to 190 °C

**Figure 7:**
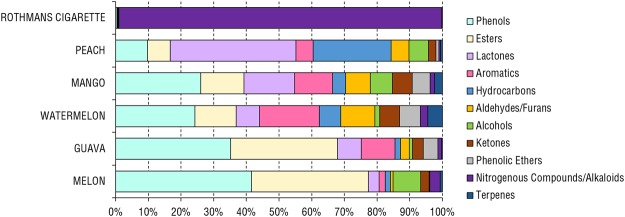
Major volatile class percentile levels in different flavored hookah tobacco products heated at 190 °C.

**Figure 8:**
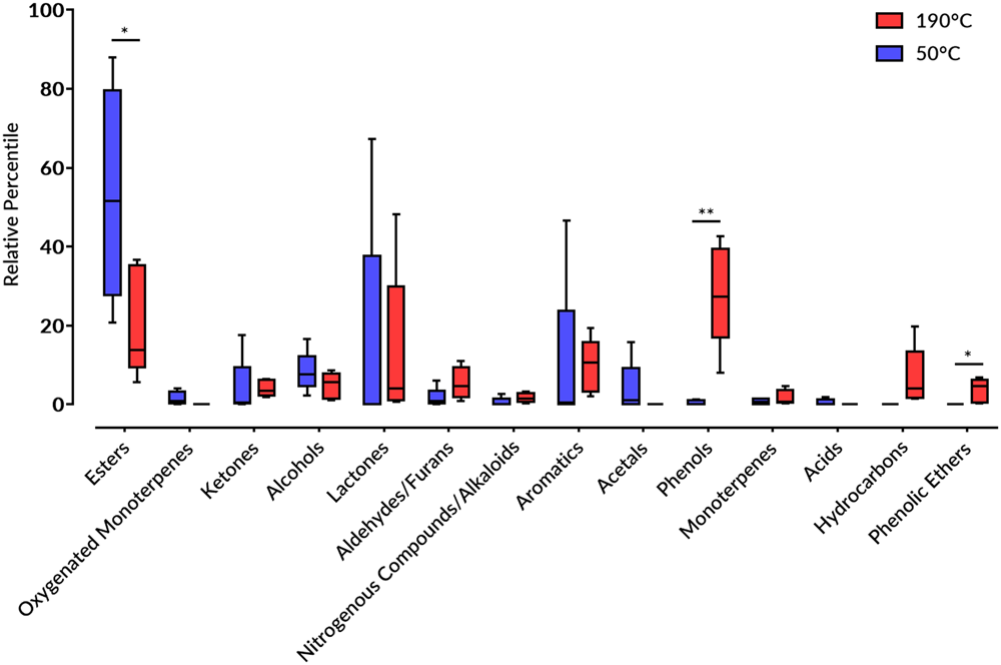
Major classes of volatiles difference between the guava, watermelon, peach, mango and melon hookah specimens treated at 50 °C and 190 °C; (*P < 0.05 and **P < 0.01).

Volatiles were collected under same methodology except for heating it up at 190 °C prior to volatiles collection from 5 flavored hookah tobacco namely, guava, watermelon, peach, mango and melon for comparison, as well as cigarette tobacco. Regarding cigarette tobacco, nicotine was undoubtedly the sole component detected at this high temperate reaching almost 99% in abundance (Fig. 7, Supplementary Table S2, Supplementary Fig. S3). For the 5 flavored hookah tobacco products, the detected volatiles profile in comprised similar classes to previous analysis, except for several phenolic ethers and aromatic hydrocarbons which were not detected before (Supplementary Table S2, Supplementary Fig. S3). On average, phenols, esters, lactones, aromatics, and hydrocarbons were the major classes present at 28%, 21%, 13%, 10% and 7%, respectively (Fig. 7). 81 new volatiles were detected in the superheated hookahs, most of which are likely to have originated from the coal tar and not the actual flavor constituents, which impose health hazard risks^63^. Figure 8 shows a comparison between the major classes of volatiles between the guava, watermelon, peach, mango and melon hookah specimens treated at 50 °C and 190 °C. The most notable class volatiles that increased with at 190 °C was phenols (*P* < 0.01) (Fig. 8).

Phenols presented the major chemical class found in the superheated hookah tobacco specimens in comparable levels in mango and watermelon flavored hookah tobacco and in higher levels in melon and guava flavored hookah tobacco. 27 different phenolic compounds, including phenol and its derivatives, such as methyl phenols (cresols), dimethyl phenols (xylenols), trimethyl phenols (pseudocumenols), and methoxy phenols (guaiacol). These chemicals are major constituents of coal tar^64^ and found to be cytotoxic^65,66^. Cresols at high concentrations can induce hepatic injury, whereas p-cresol was found to be cytotoxic to vascular endothelial and mononuclear cells^67^. Aromatics comprised 24 different volatiles made-up of benzene, naphthalene and their derivatives. Coal tar is distinct for its naphthalene-like odor, due to the abundance of naphthalene in its chemical mixture^64^. Naphthalene and its derivatives are known carcinogens and causative of haemolytic anaemia^68^, and were found in higher levels in watermelon and guava rather than other specimens. γ-Decalactone, a lactone with strong peach aroma, was found in a considerable level 12% and 5% in peach and mango hookah tobacco, respectively, and it is used for flavoring beverages and food. As well as, γ-decalactone isomer was found in peach, mango and guava hookah tobacco at 19%, 5% and 4%, respectively; and γ-undecalactone only in peach hookah tobacco at 18%. Three hydrocarbons were detected in all flavored hookah tobacco specimens, but in higher levels in peach, mango and watermelon, at this high temperature including pentadecane, tridecane and hexadecane, which were also found in cheese^69^. Tridecane was detected at a high level almost 18% in peach hookah tobacco. These results augment our hypothesis that the superheated hookah volatiles are shifted more towards monitoring degradation of coal tar constituents, of which some products are considered detrimental to human health. In light of these findings, the flavored hookah tobacco products are on the high-risk scale together with the Kas unflavored tobacco.

Finally, to establish the effect of water on volatiles profile, a representative product (mango-flavored hookah tobacco) was analyzed in the presence of water at 190 °C and results of attained volatiles were compared to previous dry analysis (Supplementary Fig. S4A) showing no qualitative differences. PCA of attained results showed no clear segregation along PC1 accounting for 40% of the total variance (Supplementary Fig. S4B). Such results show that the previous volatiles profiling analyzes for the different products can be relied on for simulating volatiles produced during hookah usage.

## Discussion

The popularity of hookah cafes is on the rise around the world^12^ and consequently many restaurants and coffee shops are now serving hookah in an effort to attract customers and promote their sites. Different shapes, colors and designs for hookah devices are manufactured in addition to the new flavors that are the main subject of promotion^12^. This study presents the first comprehensive chemical profile of, a total of 114, components emitted during hookah usage and their classification into 11 main chemical classes (Supplementary Table S1) as well as relating them to reported health hazards. Comparing chemical components of non-flavored hookah tobacco and cigarettes showed higher health hazards produced by degradation products of heated coal during hookah usage. Unlike cigarettes volatile profile, which showed mostly nicotine at high temperature, this study presents strong evidence for the presence of coal tar degradation products, mainly aromatics, in the analyzed flavored hookah tobacco products (Supplementary Table S2). Irritant components as furans, phenols, and acids, some of which are found elevated at higher temperatures in addition to carcinogenic and reported hazardous volatiles including nitrogenous compounds and different alcohols all which support the underestimated danger of smoking hookah tobacco on human health. In this study, comparing the flavor profiles of each product, one can conclude that in terms of health hazards, the melon flavored tobacco strikes as the worst so far, with 4 potentially undesirable components. As reported previously, an increase in the awareness of the health hazards of cigarette smoking has led to a decrease in its consumption^5^. Similarly, the misconception of the safety of hookah smoking and ignorance of its health risks has probably made way for the rise in its usage, thus aggravating the problem. The appeal of flavored hookahs among consumers especially adolescents poses a threat for rapid and increased usage especially when misleading labels of “herbal shisha” is used^12^. The widely spread misconception that hookah is a safer alternative to cigarette smoking is a result of less information and more false advertising^12^, which this study aims at defying. “What is in your flavored hookah tobacco?” (Fig. 1), a question that we hope grabs the attention of readers worldwide, helps raise awareness on the risks of hookah usage and forces implementation on regulation of production and consumption.

Regarding the implications of this study on legislations, following the final FDA rule in 2016 on the Family Smoking Prevention and Tobacco Control Act (FSPTCA)^70^, there was a need to improve water pipe (hookah) regulations. A better understanding of this complex device and the components involved in its consumption will no doubt aid in adopting specific adaptations of the rules imposed by the FDA in an effort to regulate its use. Building a strong scientific base on hookah tobacco product research is what legislation agencies need for any future rulemaking regarding hookah usage^12^. This study aims at providing a representation of substances that consumers are exposed to upon hookah usage. The identified flavor profiles are essential for additives regulation by the FDA and quality control of marketed hookah tobacco products.

Future work worth considering is the impact of biological factors pertaining to human saliva and/or oral microbiome on the biotransformation of hookah volatiles. Using this volatiles collection platform coupled to multivariate data analyses has potential to be used for assessing many other factors and to better determine variations underlying hazardous effects for smoking hookah in humans, although absolute quantification is also necessary to be investigated to determine the toxicity of such hazardous compounds. These factors may include changes in additives or flavor enhancers by producers, avoiding certain flavors with higher health risks based on knowledge of constituents, or even associating certain health issues with recognizable allergens or irritants in the now known product ingredients.

## Methods

### Materials

Eleven commercial flavored hookah tobacco products from different manufacturers including “Al Fakher Tobacco Trading” brand name produced in Ajman, United Arab Emirates versus “Dandash” produced in Egypt. Analyzed flavors included, apple, green grape, guava, melon, watermelon, strawberry, cinnamon, mango, peach flavored hookah tobacco, and “Kas” unflavored hookah tobacco products from Dandash Company, Mansoura, Egypt (EG). Whereas, mango, peach, licorice, cinnamon, melon and strawberry flavored hookah tobacco products from “Al Fakher Tobacco Trading” Ajman, United Arab Emirates (EM) were purchased from hookah tobacco shops. Melon and apple flavored hookah tobacco products were analyzed from both manufacturers to assess how origin affected hookah flavor volatile profile to amount to a total of 13 hookah tobacco products Rothmans tobacco cigarettes were also used in the analyzes as a control for comparison purposes.

SPME fibres of stableflex coated with divinylbenzene/carboxen/polydimethylsiloxane (DVB/CAR/PDMS, 50/30 µm) or PDMS (polydimethylsiloxane) were purchased by Supelco (Oakville, ON, Canada). All other chemicals were purchased from Sigma Aldrich (St. Louis, MO, USA).

### Processes

The HS-SPME volatile analysis was carried as stated by Farag *et al*.^71,72^ with slight modifications. For hookah flavor volatiles collection, 0.5 g of hookah tobacco material was placed inside SPME screw cap vials (20 ml) followed by the addition of 2 μg of *(Z)*-3-hexenyl acetate prepared in water as an internal standard. The SPME fiber was inserted manually into the vial containing the material and placed in an oven with shaking using magnetic stirrer and maintained at 50 °C for 30 min. The fiber was subsequently withdrawn into the needle and then injected into the injection port of the gas chromatography-mass spectrometry (GC-MS). GC-MS analysis was performed on a Shimadzu GC-17A gas chromatogram equipped with DB-5 column (30 m × 0.25 mm i.d. × 0.25 *µ*m film thickness; Supelco) and coupled to Shimadzu QP5050A mass spectrometer. The interface and the injector temperatures were both set at 220 °C. The following gradient temperature program was used for volatiles analysis. The oven temperature was kept first at 40 °C for 3 min, then increased to 180 °C at a rate of 12 °C min^−1^, kept at 180 °C for 5 min, and finally ramped at a rate of 40 °C min^−1^ to 240 °C and kept at this temperature for 5 min. The carrier gas Helium was used at a total flow rate of 0.9 ml/min. Blank runs were made during samples analyzes using only charcoal. The HP quadruple mass spectrometer was operated in EI mode at 70 eV. A scan range was set at the ratio of mass to charge number of ions (*m/z)* 40–500. For heated hookah flavor volatiles collection, 0.5 g of hookah material was mixed with 0.5 g heated charcoal inside SPME screw cap vials (20 ml) placed on a hot plate kept at 190 °C for 10 minutes followed by the addition of 2 ml distilled water. Water was added to mimic hookah vapors exposure as volatiles are filtered in water prior to inhalation. 2 μg of *(Z)*−3-Hexenyl acetate prepared in water was then added as an internal standard, SPME fiber was inserted manually into the vial containing the material and volatiles were collected and analyzed under the same conditions as described above. Peak normalization was carried out in reference to spiked (*Z*)-3-hexenyl acetate, added to all flavors prior to volatiles analysis.

### Volatiles identification and multivariate data analyses

Volatile components were identified by comparing their retention indices (RI) relative to n-alkanes (C6–C20), mass matching to the National Institute of Standards and Technology (NIST) mass spectral library, WILEY library database and with standards whenever available. Matching library spectra above a threshold of 90% with regard to MS1 and MS/MS level was considered acceptable. Peaks were first deconvoluted using AMDIS software (www.amdis.net) prior to mass spectral matching. Volatiles abundance data were prepared for multivariate data analysis by extraction using MET-IDEA software^73^ for data extraction. Data were then subjected to principal component analysis (PCA), hierarchical clustering analysis (HCA), partial least squares-discriminant analysis (OPLS-DA) using SIMCA-P version 13.0 software package (Umetrics, Umea, Sweden). Markers were subsequently identified by analysing the S-plot, which was declared with covariance (p) and correlation (pcor). All variables were mean centred and scaled to Pareto variance.

## Electronic supplementary material

Supplementary Figures

Supplementary Tables

Supplementary Spreadsheet
