# Supplementary material for: The characterization of flavored hookahs aroma profile and in response to heating as analyzed via headspace solid-phase microextraction (SPME) and chemometrics
Source: Sci Rep. 2018 Nov 19;8:17028. doi: 10.1038/s41598-018-35368-6 (PMC6242864; doi:10.1038/s41598-018-35368-6)
Supplement: Supplementary file 1 — Supplementary Figures [file 41598_2018_35368_MOESM1_ESM.docx]

**The characterization of flavored hookahs aroma profile and in response to heating as analyzed *via* headspace solid-phase microextraction (SPME) and chemometrics**

**Mohamed A. Farag**^ab*^**, Moamen M. Elmassry**^c^, **Sherweit El-Ahmady**^d^

^a*^ Pharmacognosy Department, Faculty of Pharmacy, Cairo University, Cairo, Egypt

^b^ Department of Chemistry, School of Sciences & Engineering, The American University in Cairo, New Cairo 11835, Egypt.

^c^ Department of Biological Sciences, Texas Tech University, Lubbock, TX, USA

^d^ Pharmacognosy Department, Faculty of Pharmacy, Ain Shams University, Cairo, Egypt

^*^Corresponding author: Mohamed A. Farag, email: [mohamed.farag@pharma.cu.edu.eg](mailto:mohamed.farag@pharma.cu.edu.eg)

**Supplementary Fig. S1** Coefficient of variation (CV), or relative standard deviation (RSD) of all volatiles for each tobacco product to show the variability between the replicates.


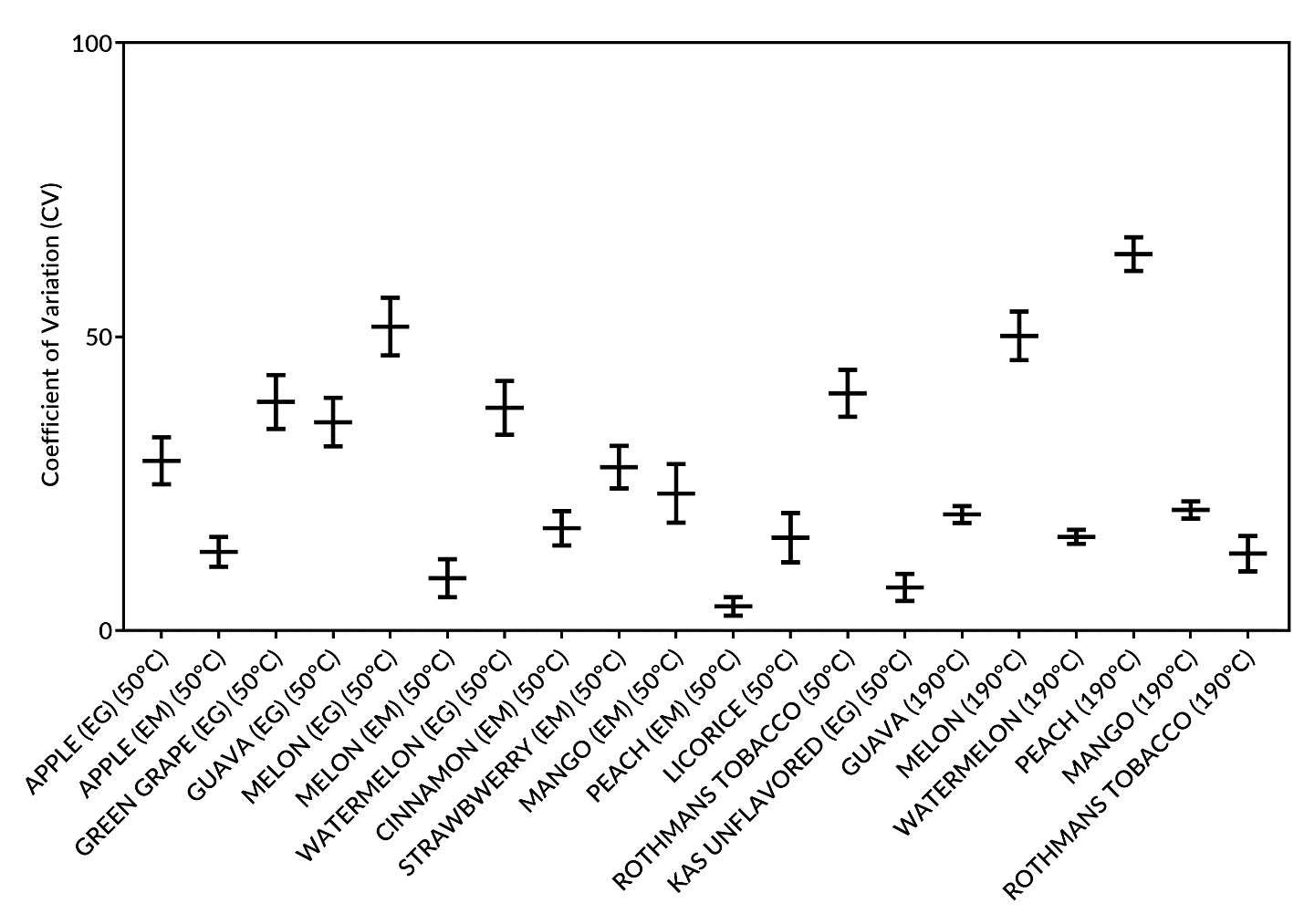


**Supplementary Fig. S2** Details of OPLS-DA models generated for each flavored hookah type against all other flavors (1A-1L) and the validation of each model.

**
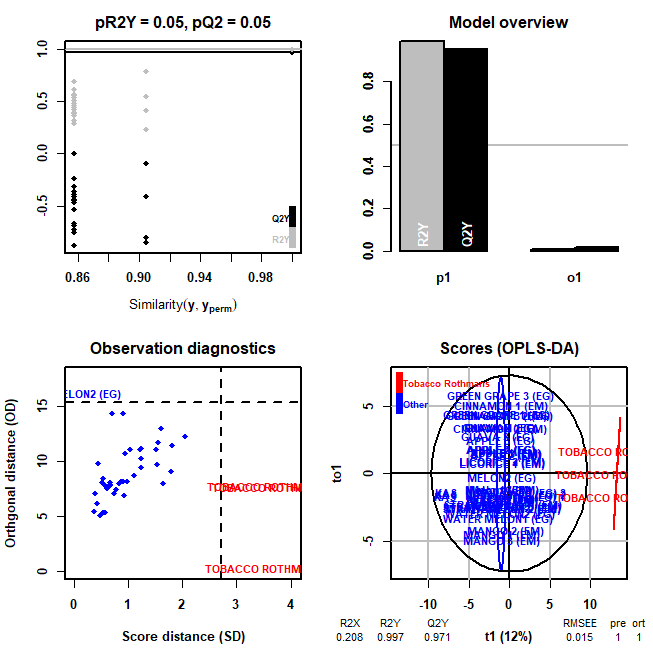
**

**
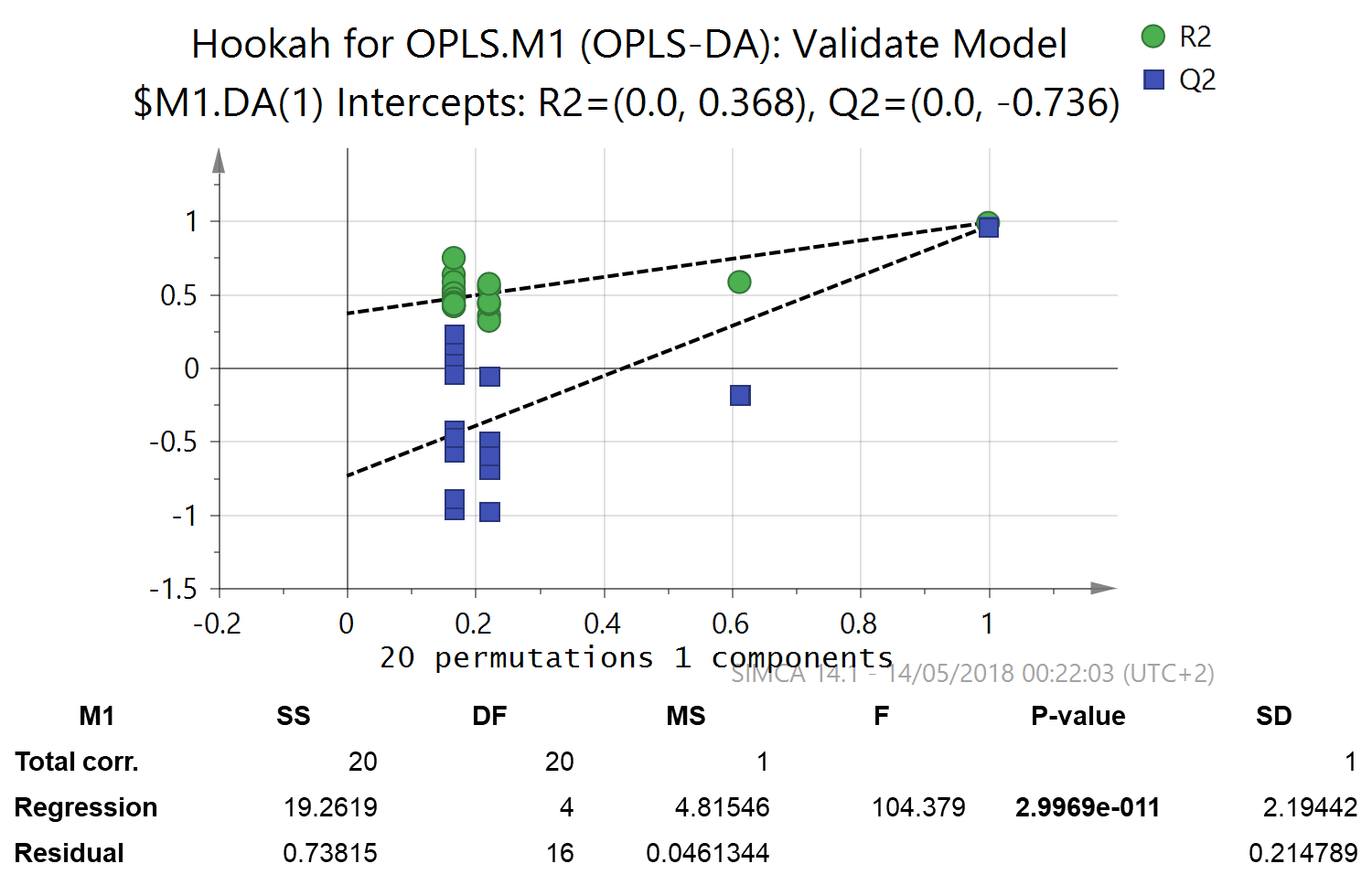
**

Supplementary Fig. S2A OPLS-DA model validation for Tobacco Rothmans against other hookah flavors


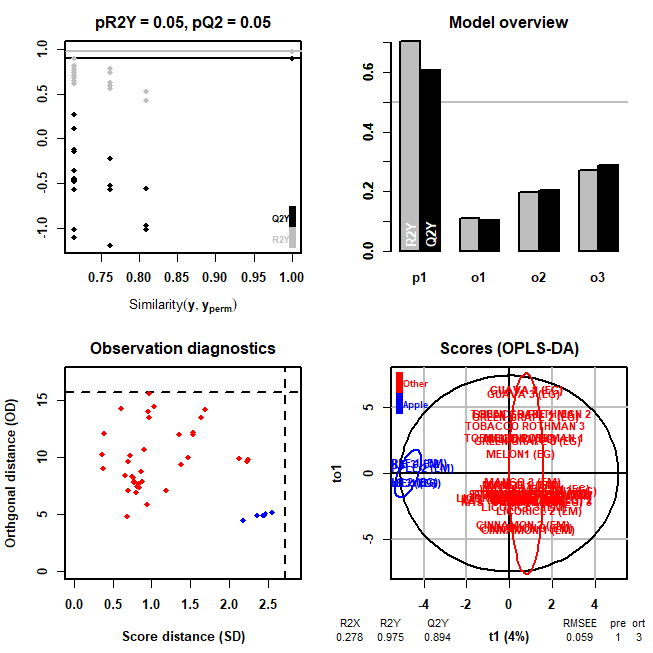


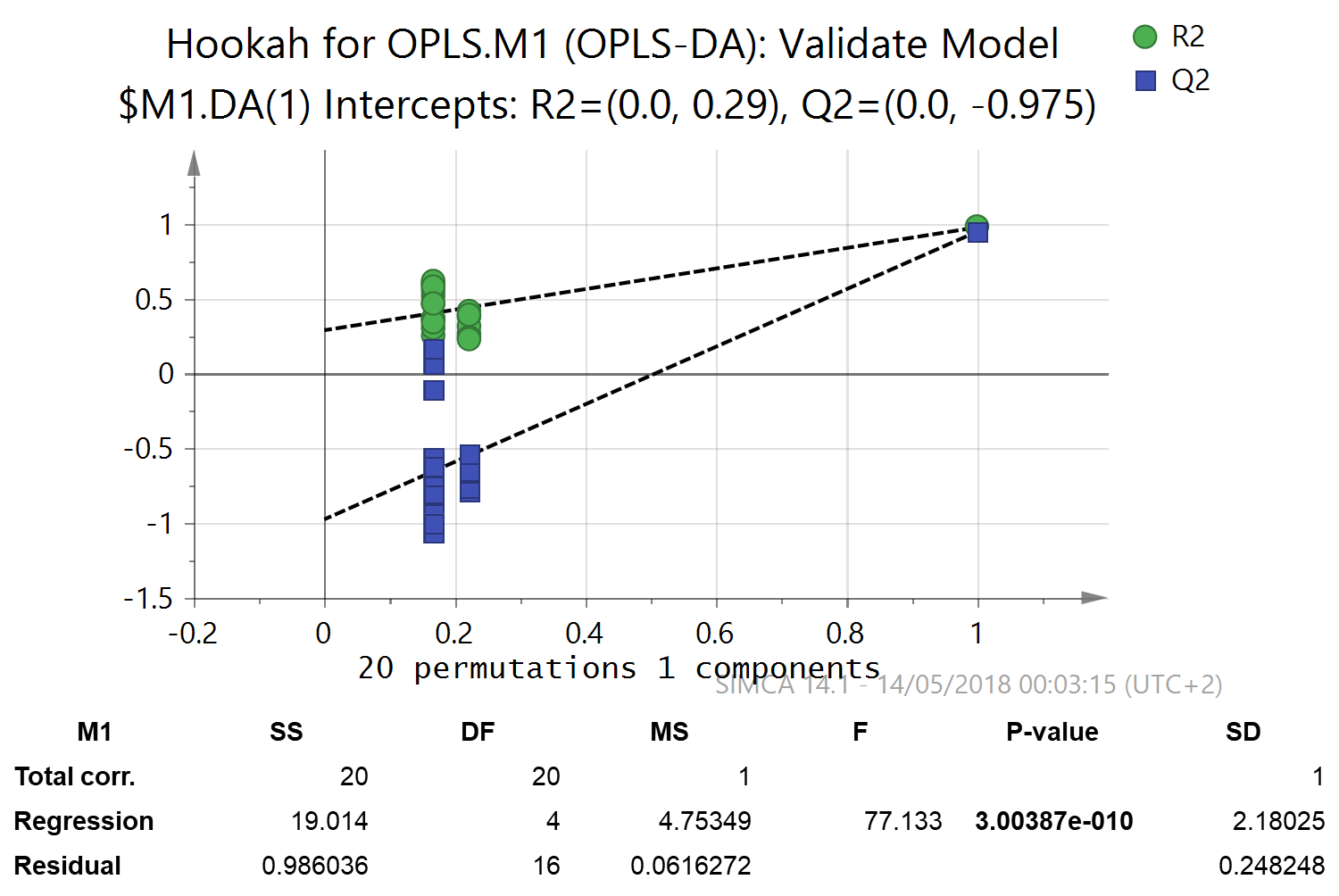


Supplementary Fig. S2B OPLS-DA model validation for Apple flavor against other hookah flavors


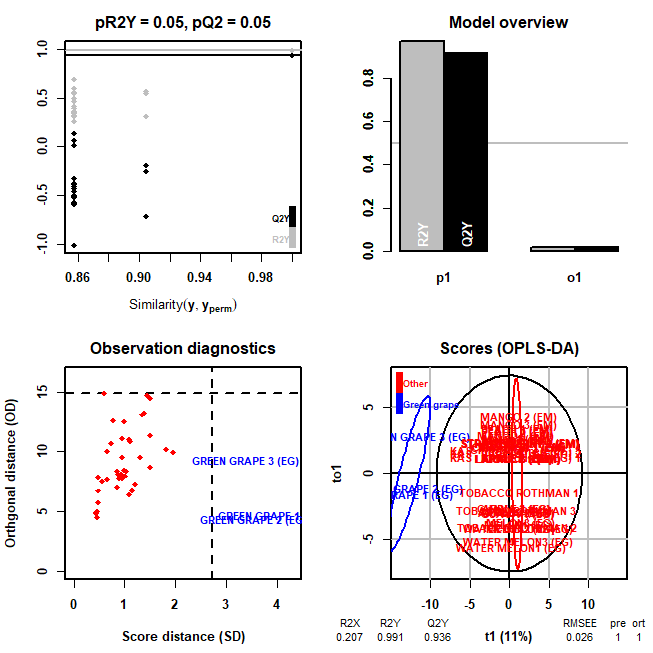


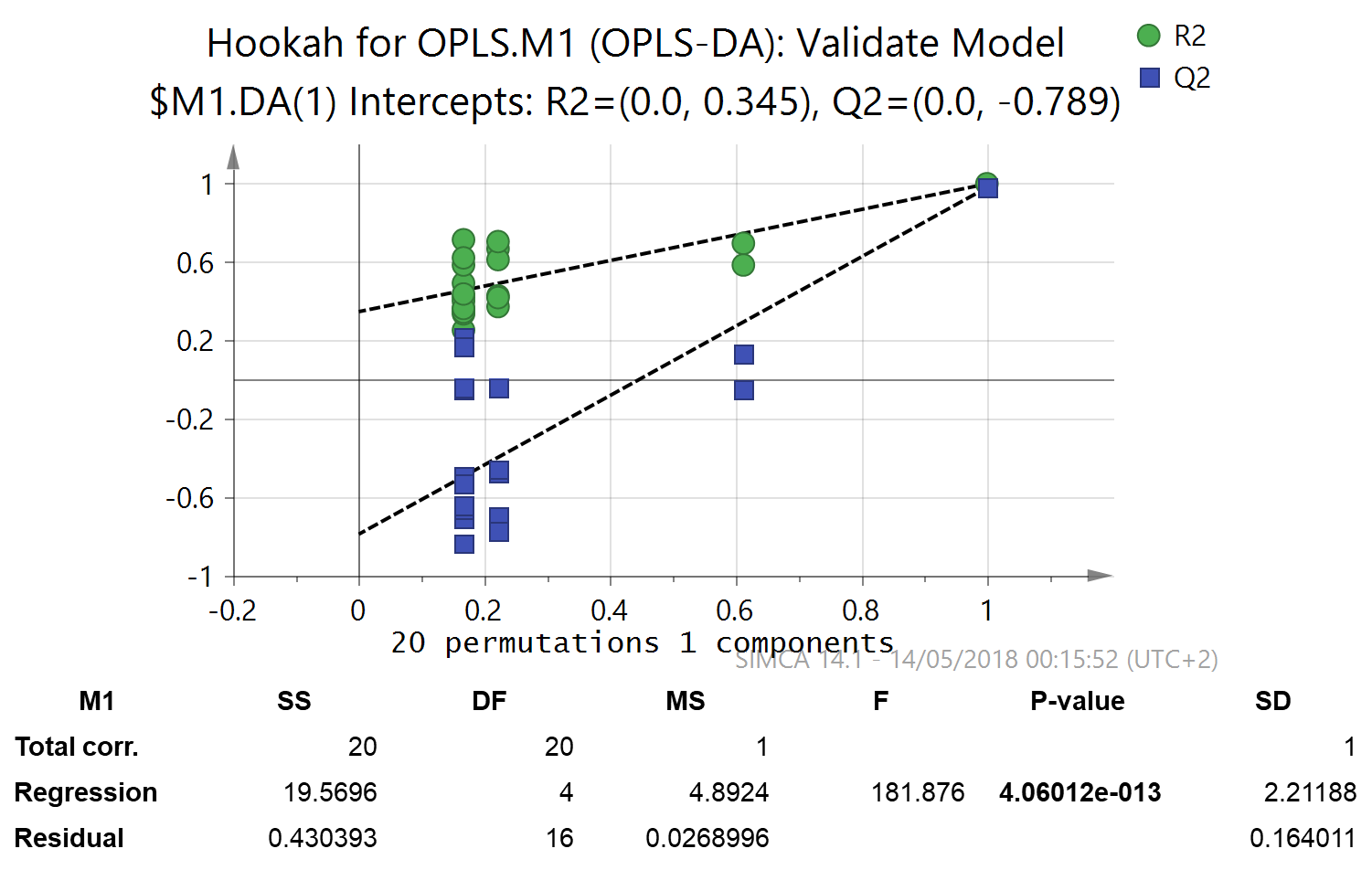


Supplementary Fig. S2C OPLS-DA model validation for Green Grape flavor against other hookah flavors


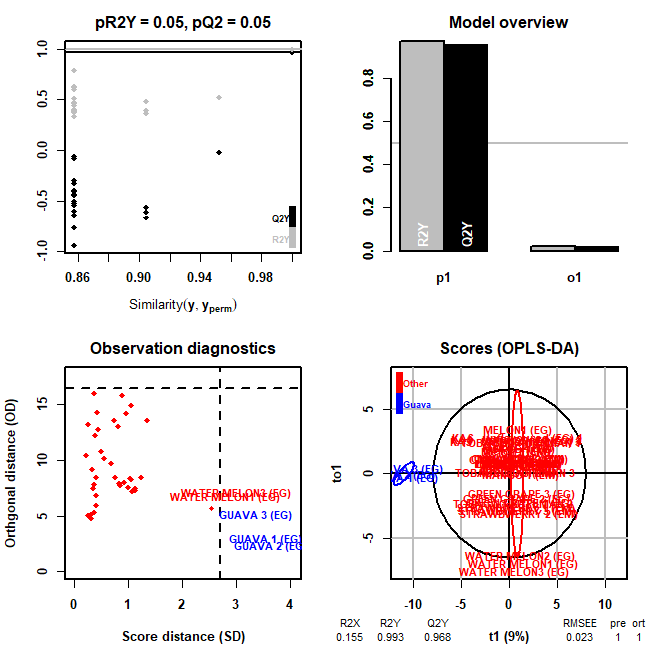


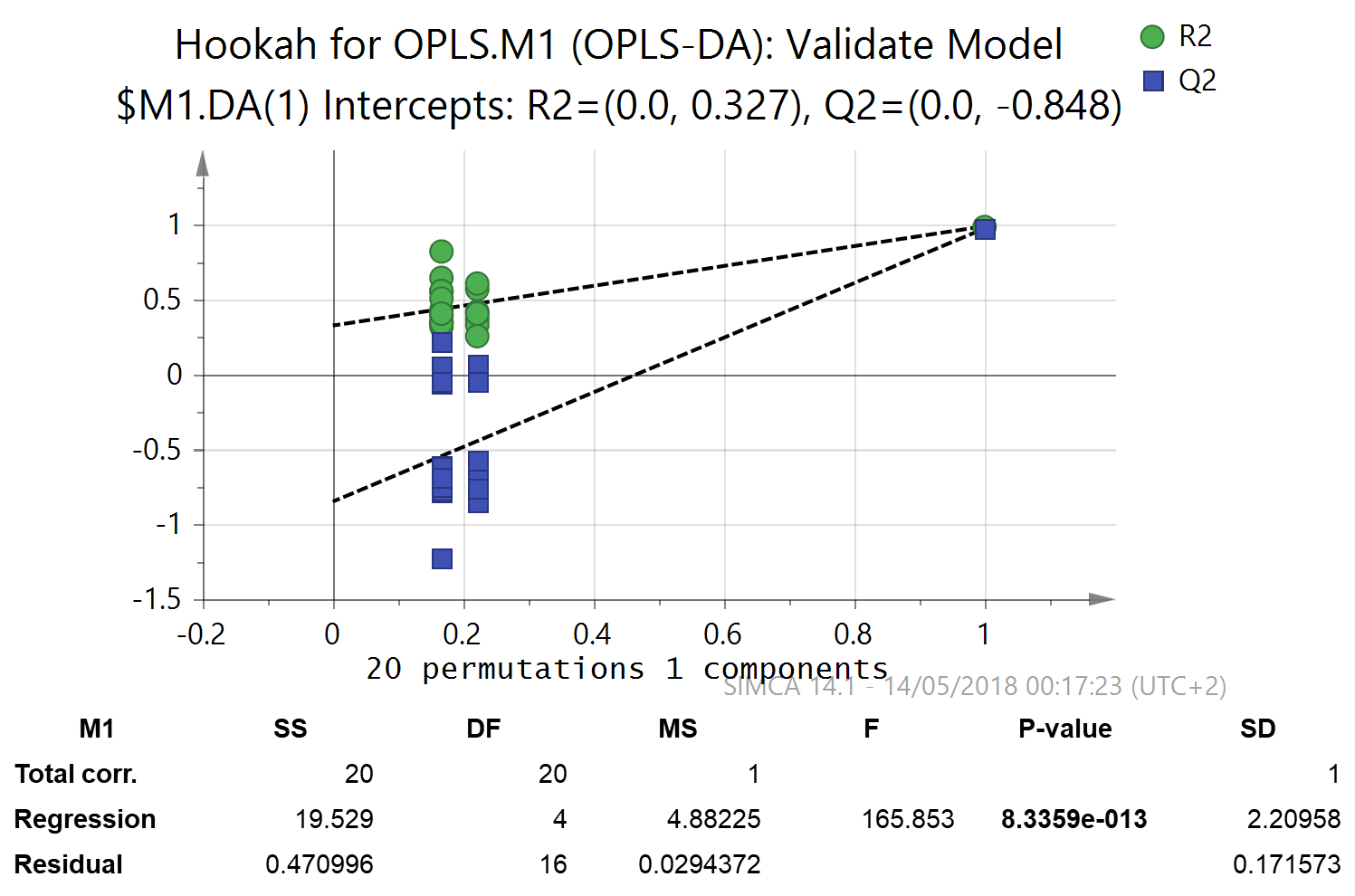


Supplementary Fig. S2D OPLS-DA model validation for Guava flavor against other hookah flavors


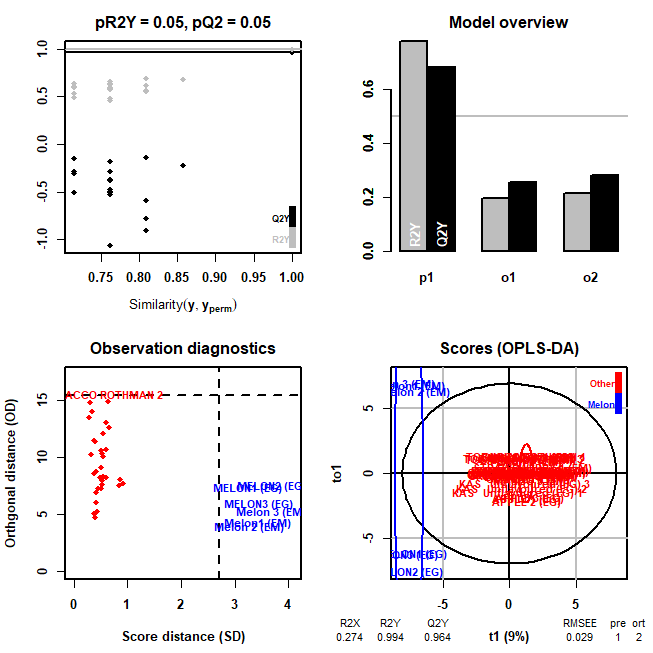


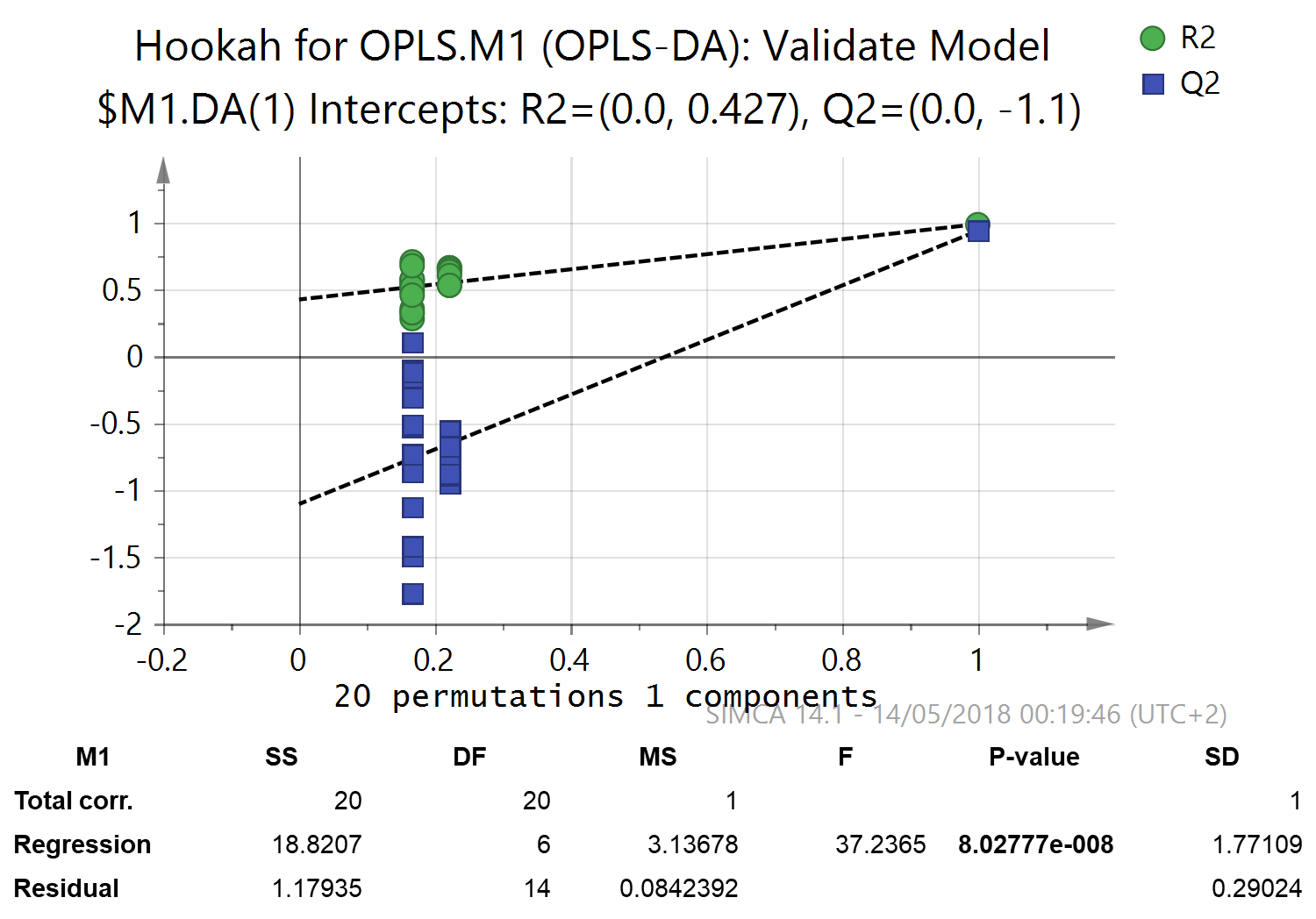


Supplementary Fig. S2E OPLS-DA model validation for Melon flavor against other hookah flavors


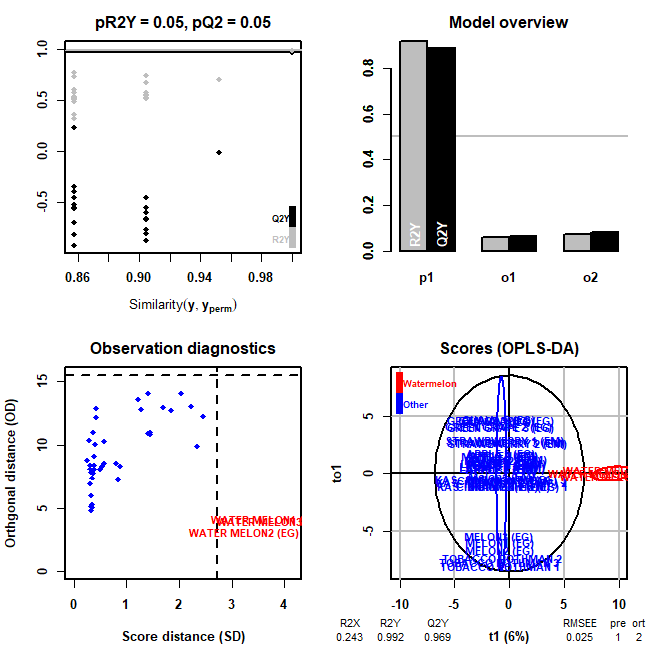


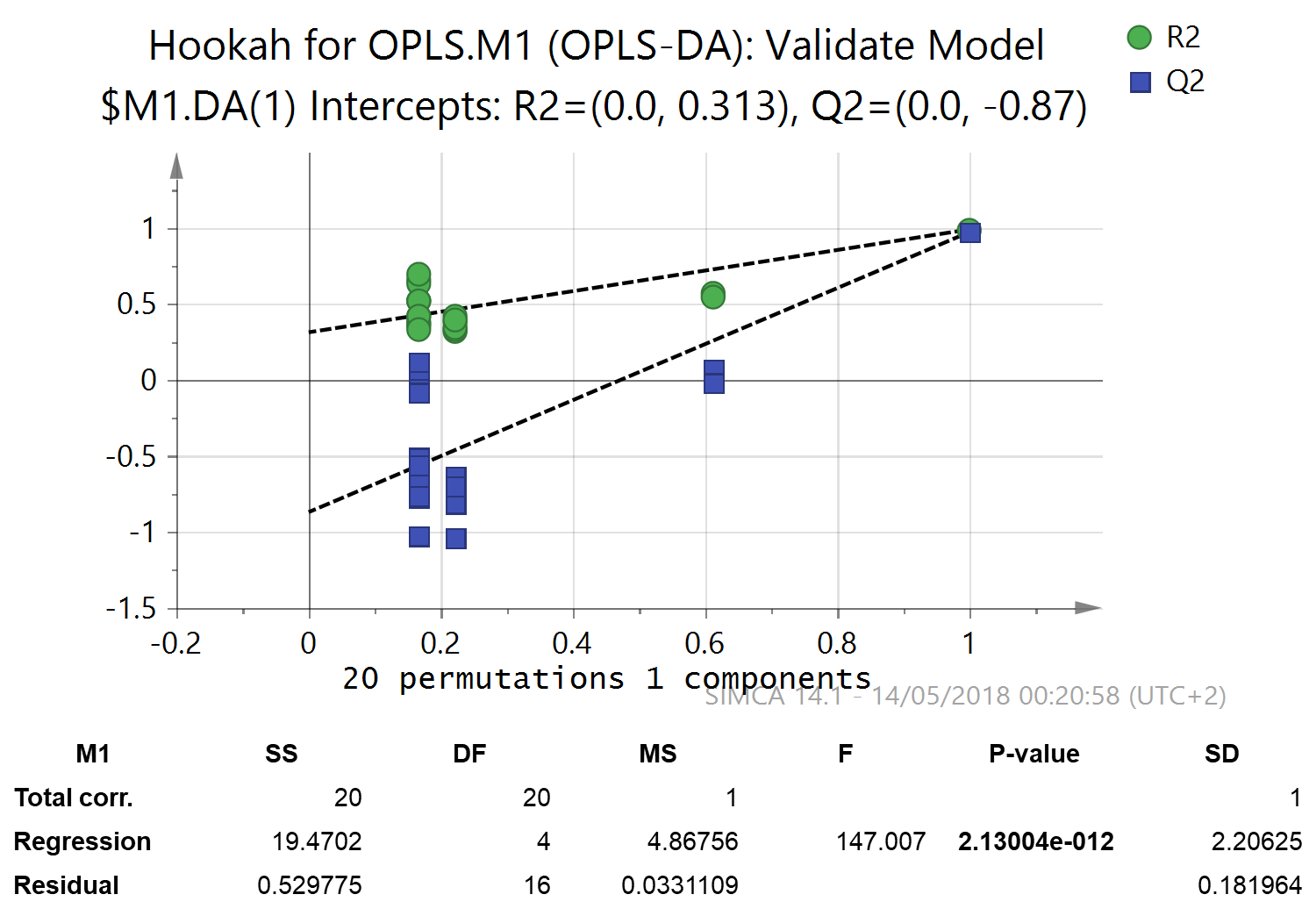


Supplementary Fig. S2F OPLS-DA model validation for Watermelon flavor against other hookah flavors


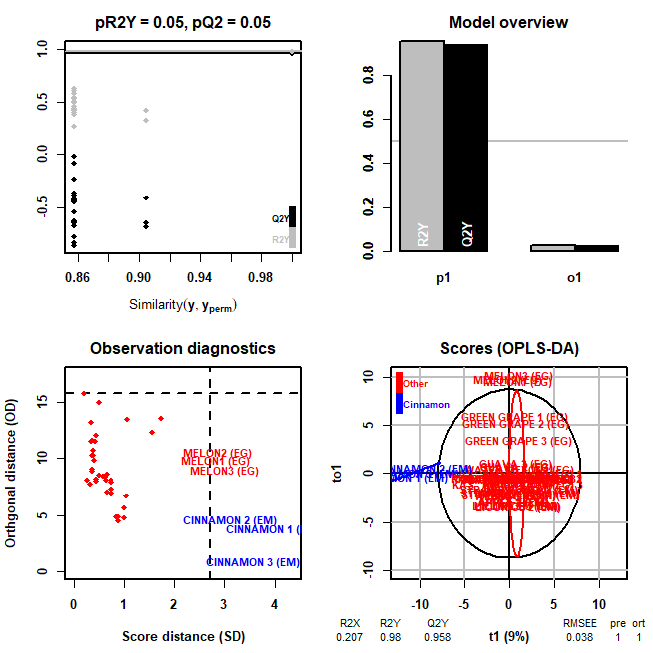


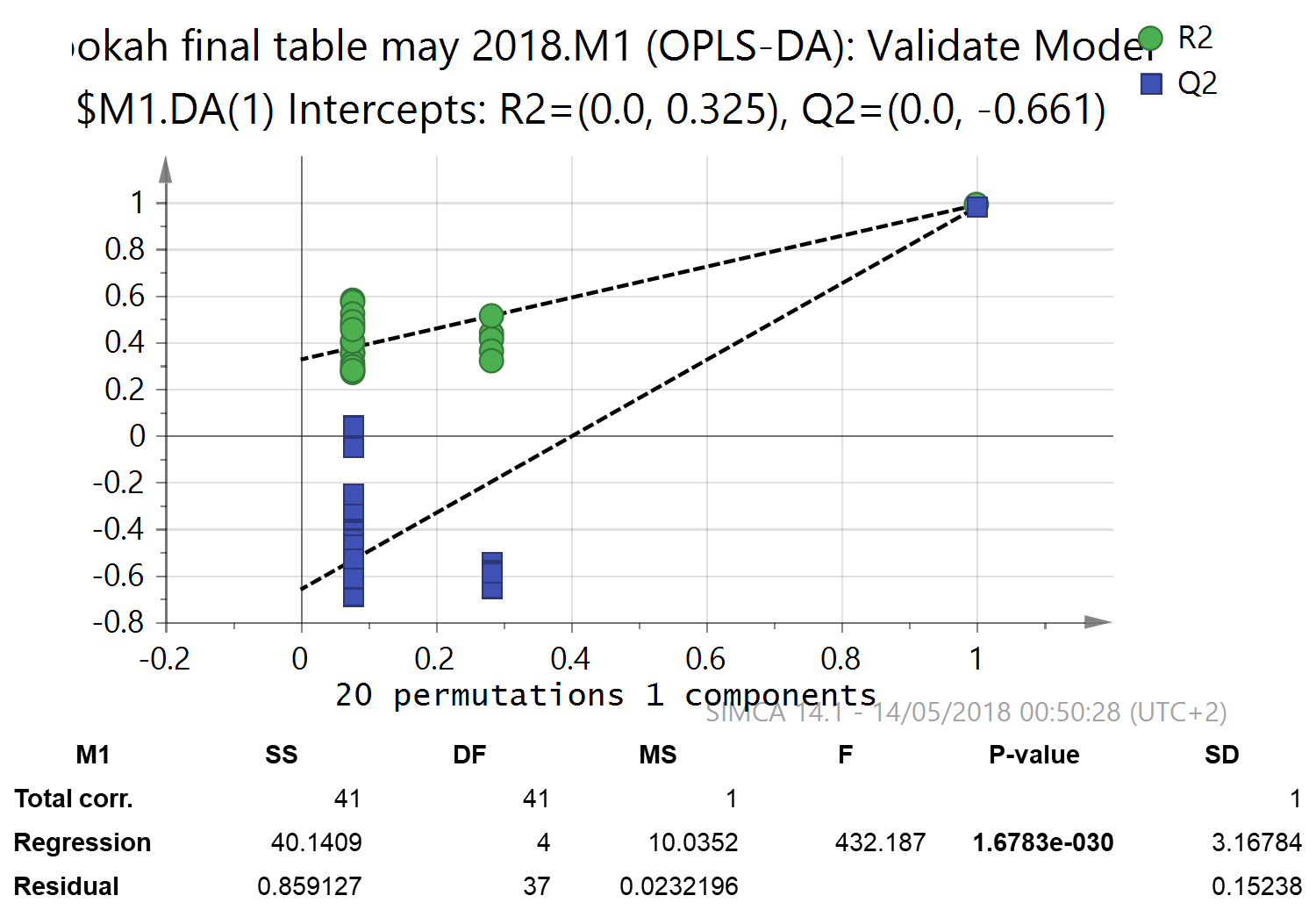


Supplementary Fig. S2G OPLS-DA model validation for Cinnamon flavor against other hookah flavors


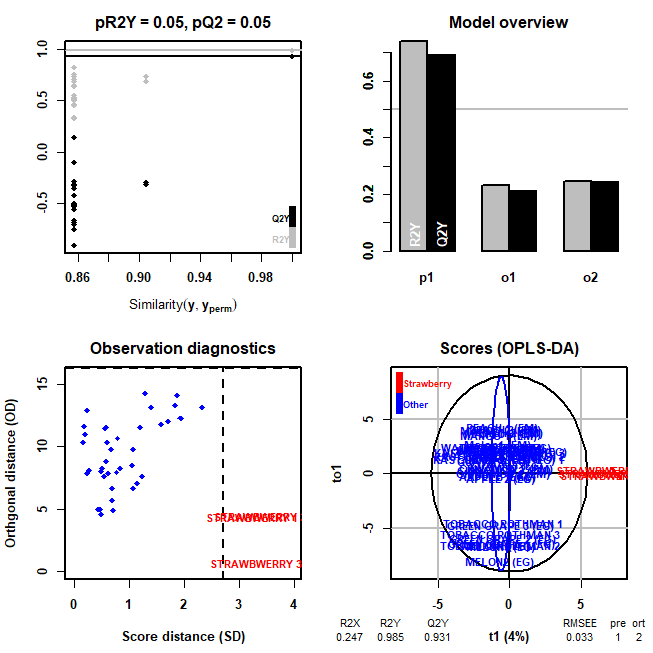


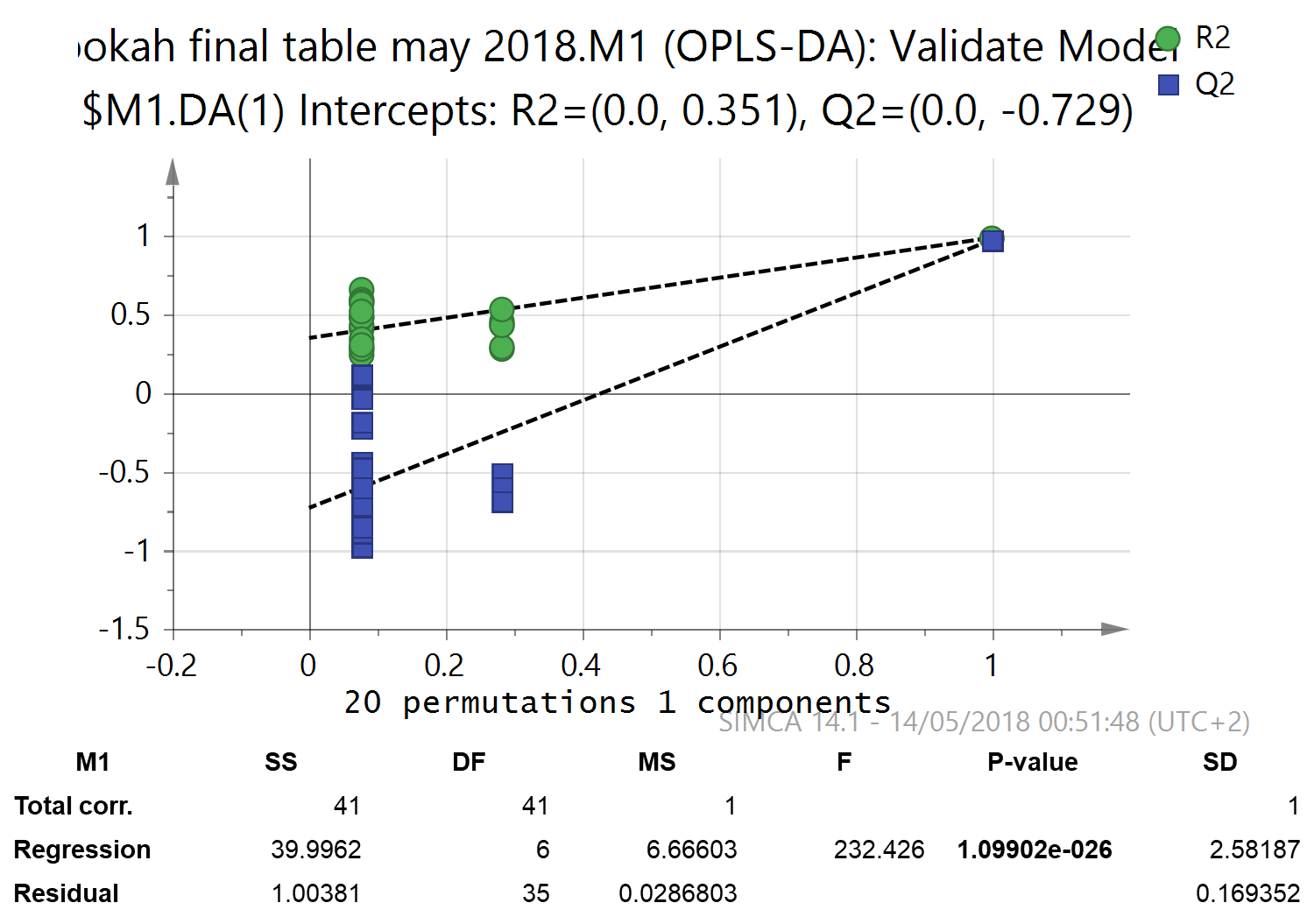


Supplementary Fig. S2H OPLS-DA model validation for Strawberry flavor against other hookah flavors


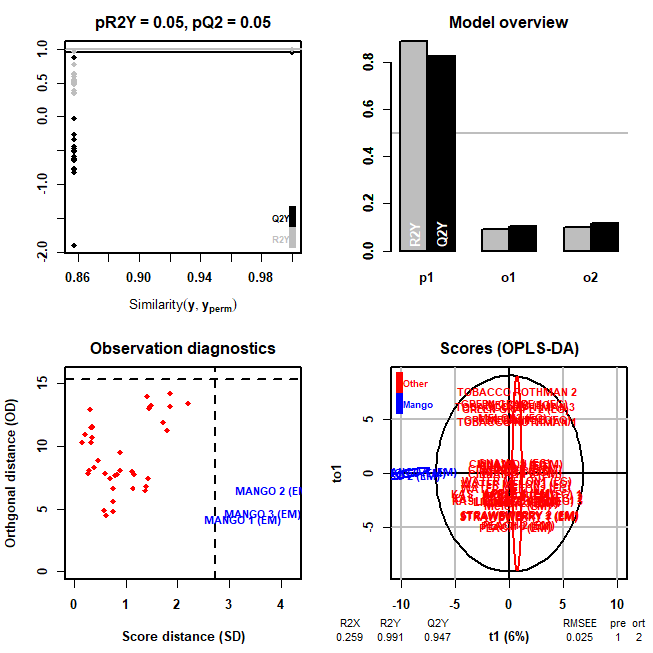


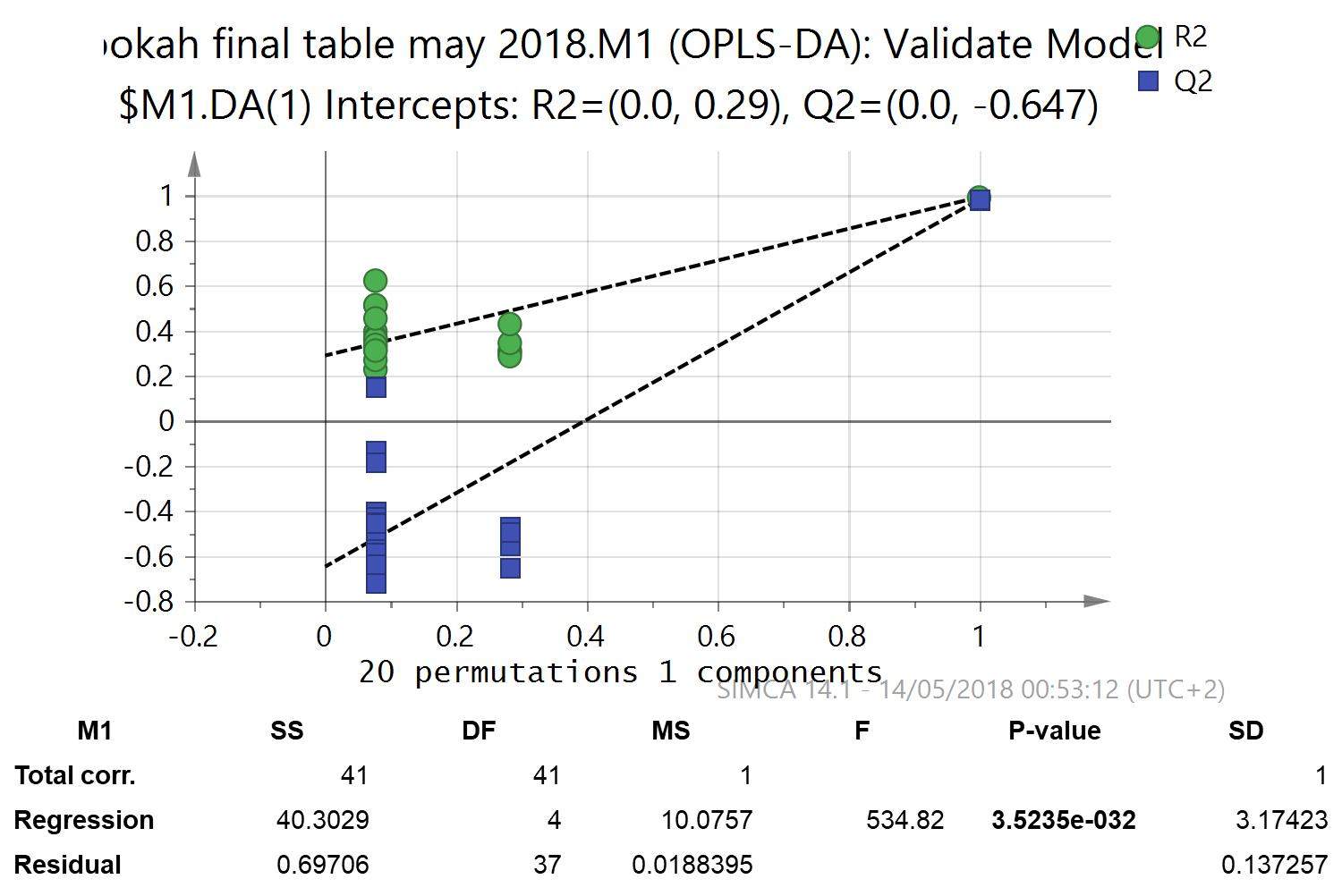


Supplementary Fig. S2I OPLS-DA model validation for Mango flavor against other hookah flavors


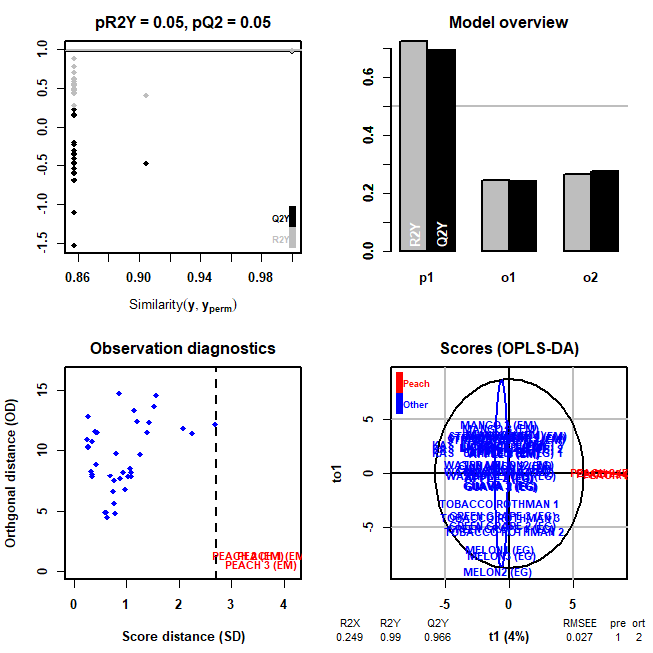


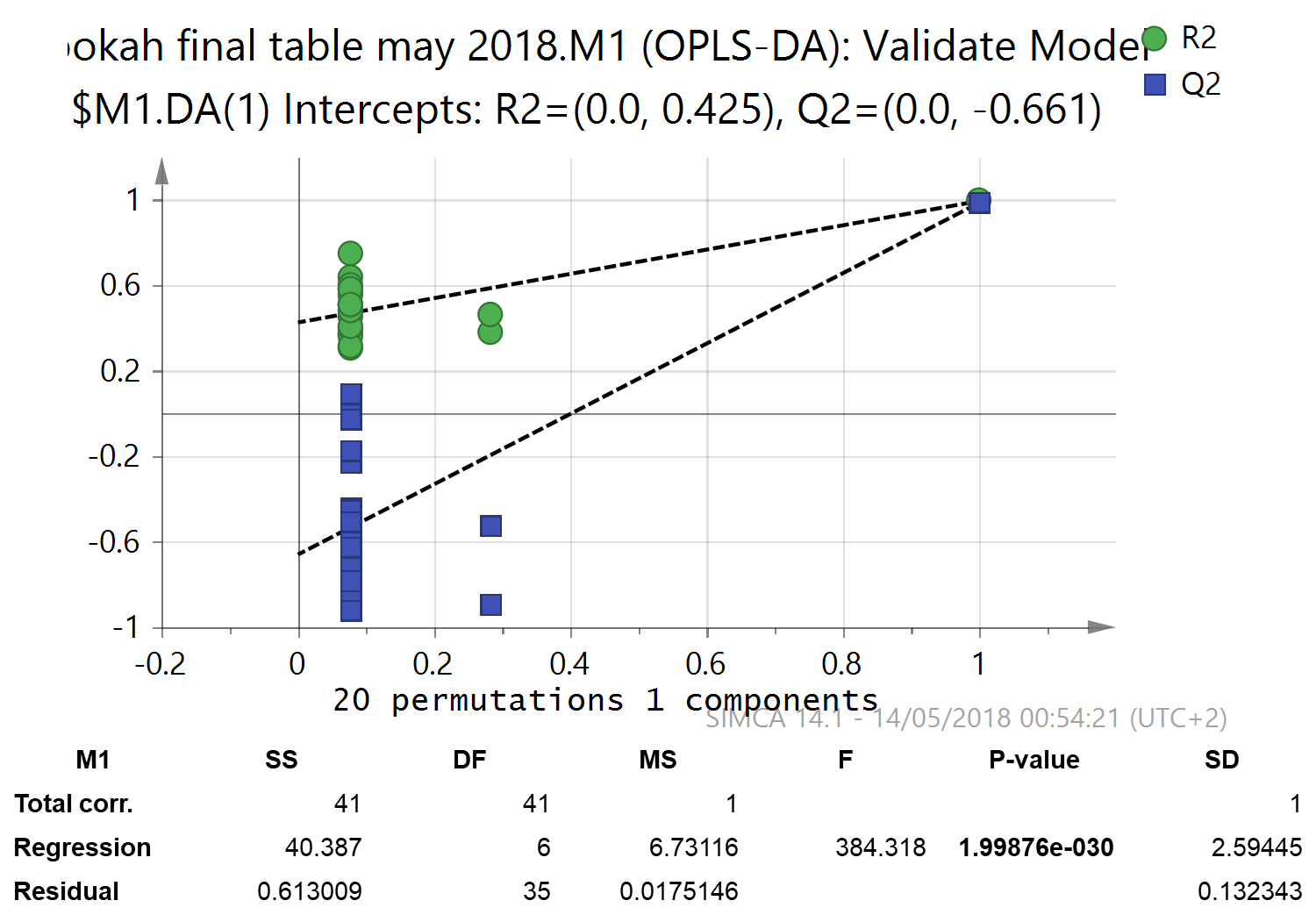


Supplementary Fig. S2J OPLS-DA model validation for Peach flavor against other hookah flavors


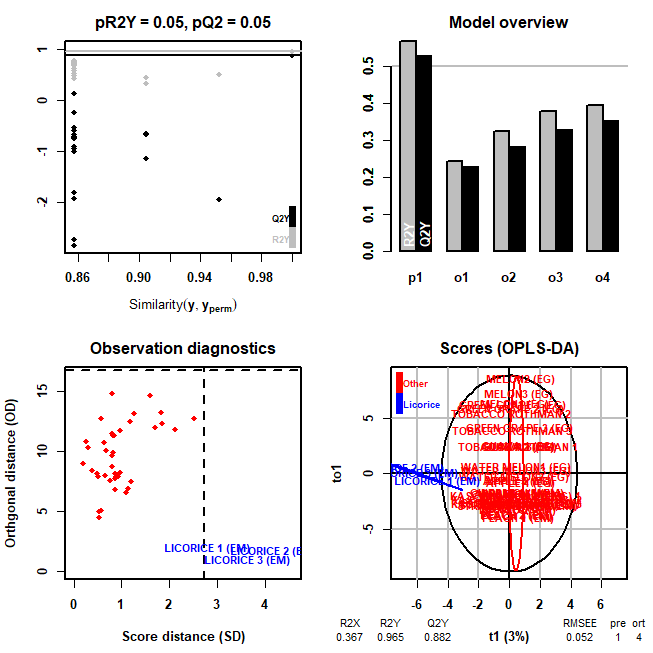


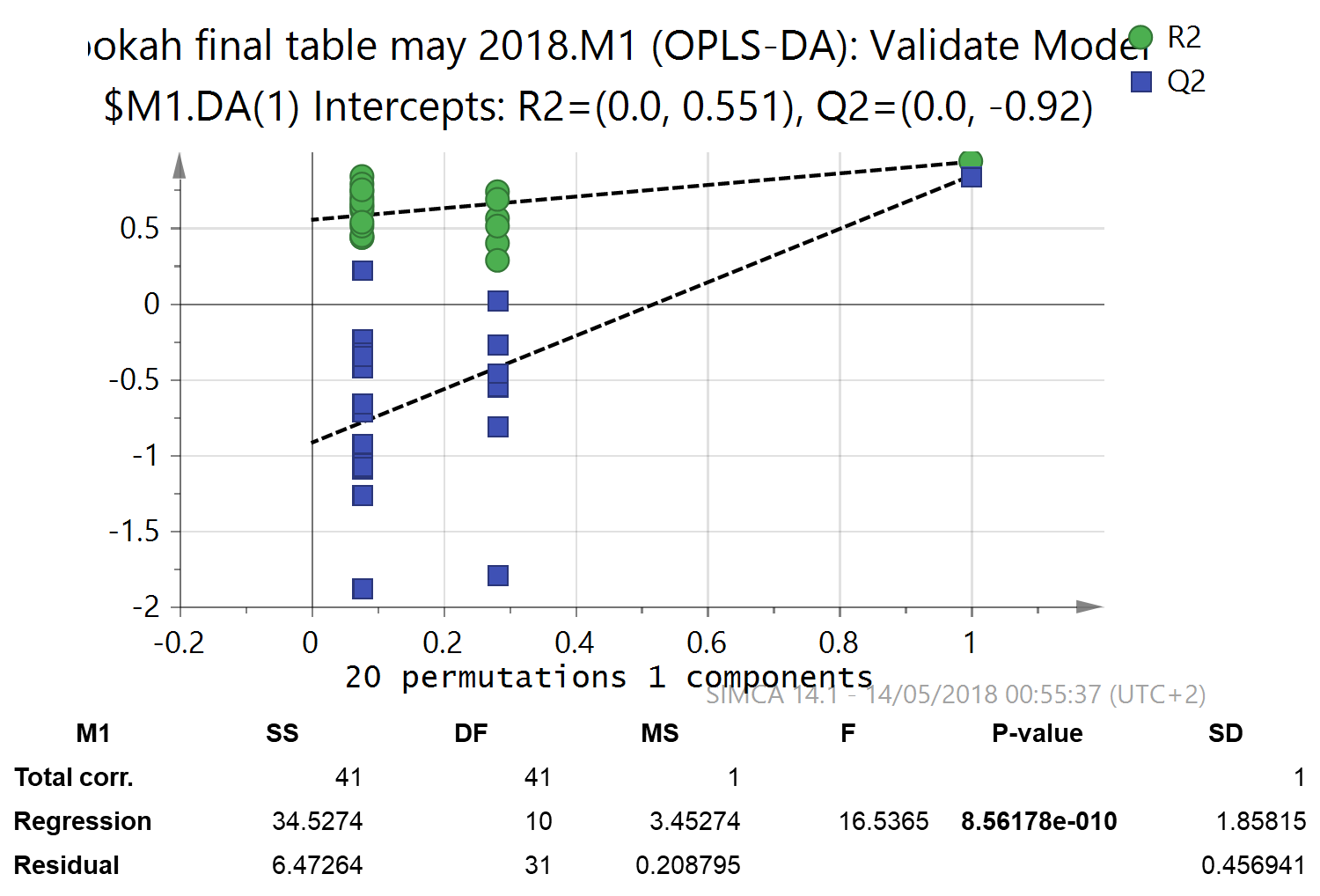


Supplementary Fig. S2K OPLS model validation for Licorice flavor against other hookah flavors


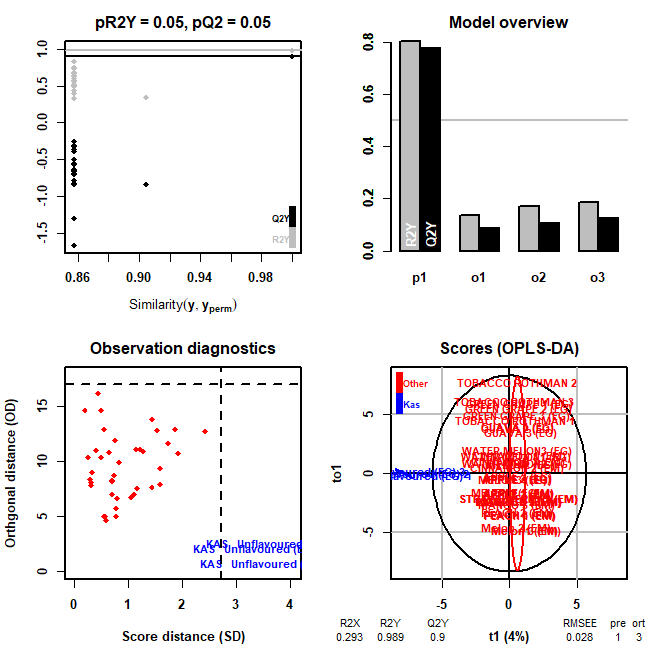


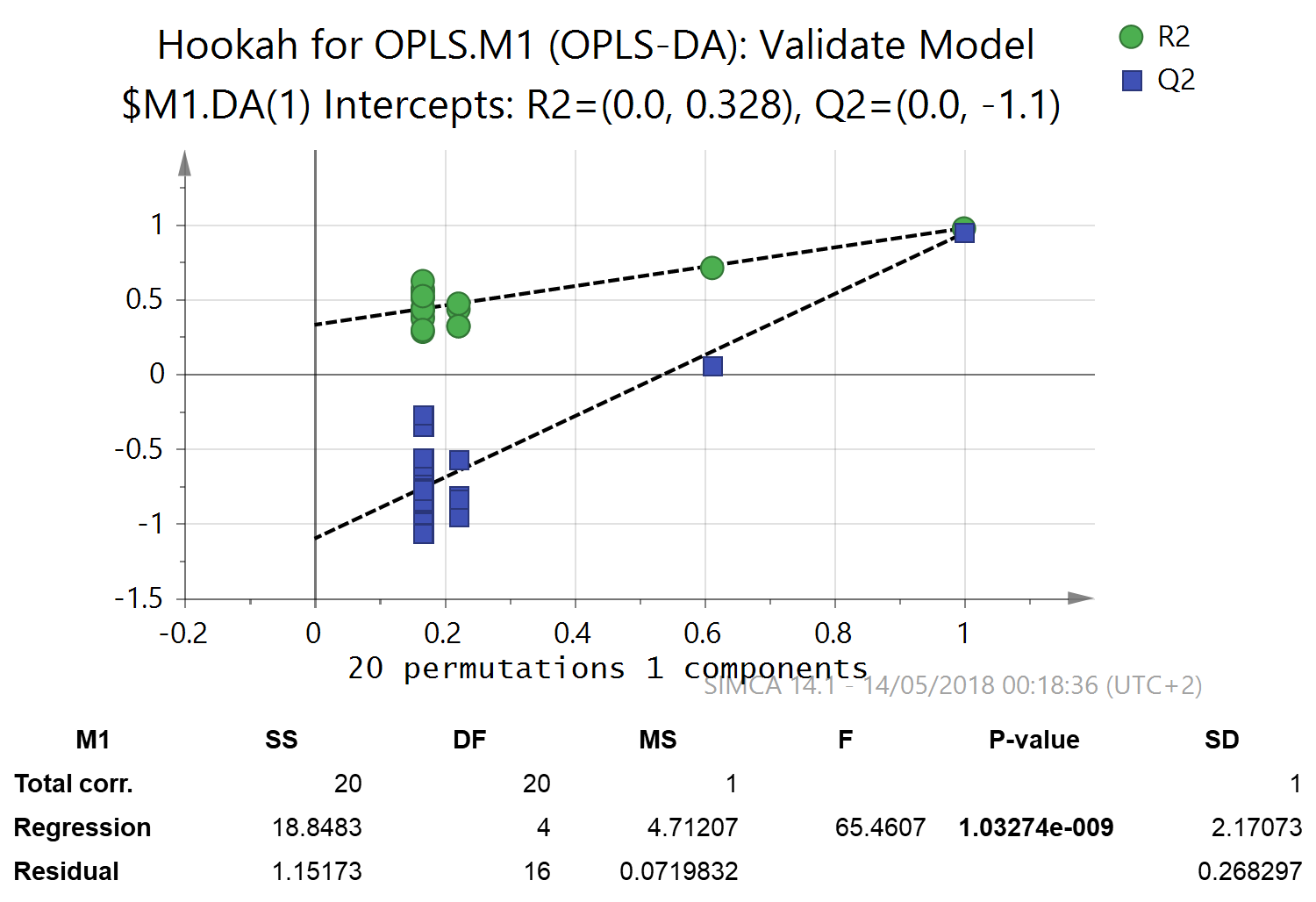


Supplementary Fig. S2L OPLS-DA model validation for Kas Unflavored against other hookah flavors

**
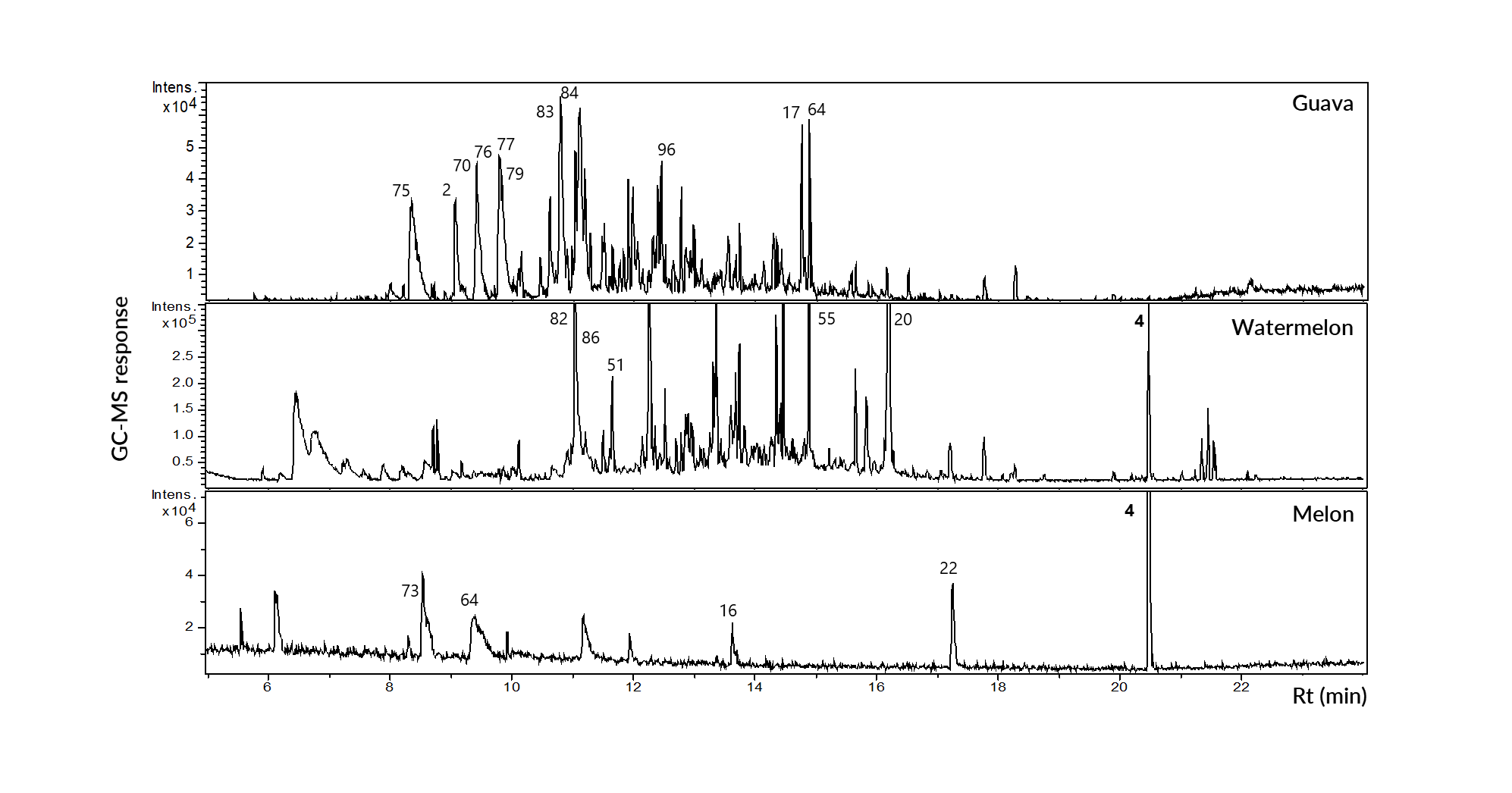
**

**Supplementary Fig. S3** SPME-GC-MS representative chromatogram of headspace volatiles collected from guava, watermelon and melon flavored hookah tobacco specimens that are heated at 190°C. The corresponding compound names for volatile peaks follow that listed in supplementary table S2. **2**, Benzyl alcohol; **4**, Tetradecamethylene glycol; **16**, *(E)*-2-Hexenyl caproate; **17**, Ethyl cinnamate; **20**, Cinnamyl isobutyrate; **22**, α-Amylcinnamaldehyde; **51**, 4,7-Dimethylbenzofuran; **55**, Pentadecane; **64**, 2,4-Dimethyl-1,3-cyclopentanedione; **70**, γ-Decalactone; **73**, N,N'-Dimethylthiourea; **75**, Phenol; **76**, o-Cresol; **77**, p-Cresol; **79**, o-Guaiacol; **83**, p-Xylenol; **84**, 3,5-Xylenol; **82**, p-Ethylphenol; **86**, o-Ethylphenol isomer; **96**, o-Acetylphenol.

**
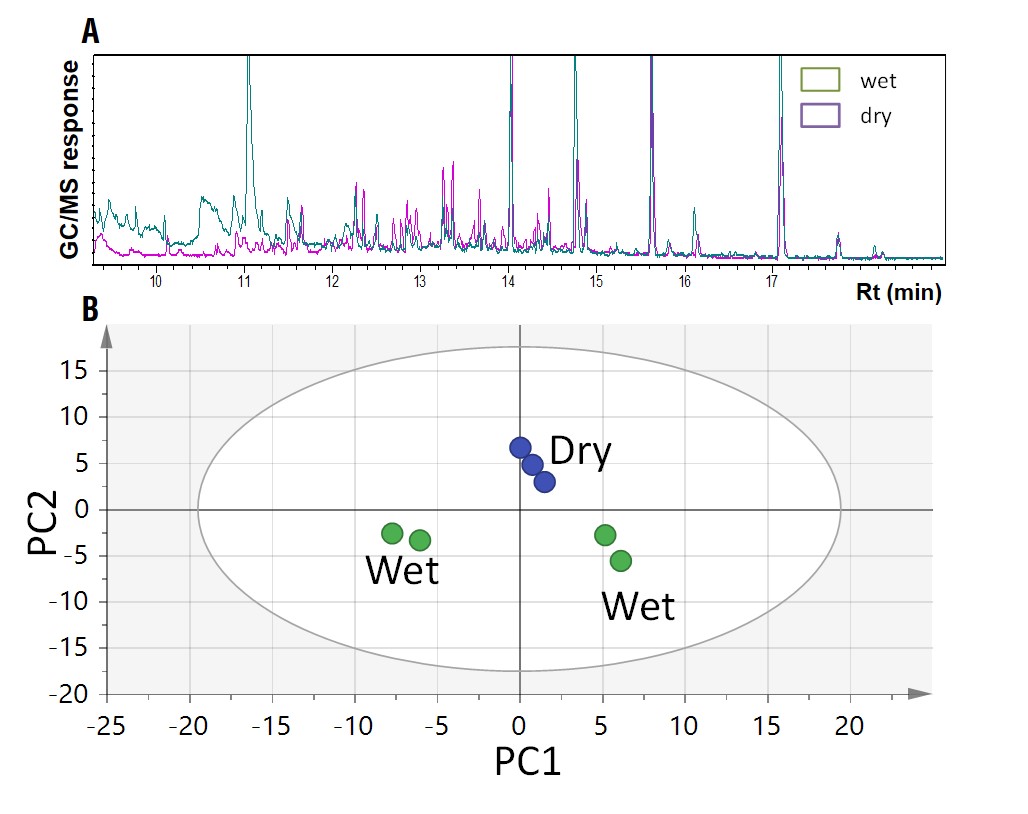
**

**Supplementary Fig. S4** Assessment of volatiles profile collected from flavored mango (EM) hookah heated at 190°C in the presence (wet) and absence of water (dry) conditions. (A) Overlaid chromatogram showing volatiles collected from flavored mango hookah heated at 190°C in dry conditions and in presence of water (wet). (B) PCA score plot analysis of SPME extracted volatile collected from flavored mango hookah tobacco heated at 190°C in presence and absence of water and with no clear specimens segregation belonging to each type along PC1 accounting for 40% of the total variance.
