# Supplementary material for: The characterization of flavored hookahs aroma profile and in response to heating as analyzed via headspace solid-phase microextraction (SPME) and chemometrics
Source: Sci Rep. 2018 Nov 19;8:17028. doi: 10.1038/s41598-018-35368-6 (PMC6242864; doi:10.1038/s41598-018-35368-6)
Supplement: Supplementary file 2 — Supplementary Tables [file 41598_2018_35368_MOESM2_ESM.docx]

**The characterization of flavored hookahs aroma profile and in response to heating as analyzed *via* headspace solid-phase microextraction (SPME) and chemometrics**

**Mohamed A. Farag** ^ab*^**, Moamen M. Elmassry**^c^, **Sherweit El-Ahmady**^d^

^a*^Pharmacognosy Department, Faculty of Pharmacy, Cairo University, Cairo, Egypt

^b^Department of Chemistry, School of Sciences & Engineering, The American University in Cairo, New Cairo 11835, Egypt.

^c^ Department of Biological Sciences, Texas Tech University, Lubbock, TX, USA

^d^ Pharmacognosy Department, Faculty of Pharmacy, Ain Shams University, Cairo, Egypt

^*^Corresponding author: Mohamed A. Farag, email: [mohamed.farag@pharma.cu.edu.eg](mailto:mohamed.farag@pharma.cu.edu.eg)

**Supplementary Table S1** Relative percentage of volatile components detected in the volatile blend of 13 hookah tobacco products and 1 cigarette tobacco product as analysed using SPME-GC-MS (n = 3).

| **Peak Number** | **Rt** | **KI** | **Compound Name** | **APPLE (EG)** | **APPLE (EM)** | **GREEN GRAPE (EG)** | **GUAVA (EG)** | **KAS Unflavored (EG)** | **MELON (EG)** | **MELON (EM)** | **WATERMELON (EG)** | **CINNAMON (EM)** | **STRAWBERRY (EM)** | **MANGO (EM)** | **PEACH (EM)** | **LICORICE (EM)** | **ROTHMANS CIGARETTE** |
| --- | --- | --- | --- | --- | --- | --- | --- | --- | --- | --- | --- | --- | --- | --- | --- | --- | --- |
|  |  |  |  | Average (Standard Deviation) | | | | | | | | | | | | | |
| **Total acetals** | | | | **1.31** | **0.17** | **0.3** | **15.82** | **-** | **0.11** | **-** | **2.82** | **1.38** | **0.33** | **1** | **-** | **-** | **0.26** |
| 1 | 10.933 | 1145 | Unknown acetal | 5  (0) | - | - | 15.49  (3.38) | - | tr. ^b^ | - | 1.55  (0.56) | - | 0.07  (0.05) | tr. | - | - | - |
| 2^a^ | 11.142 | 1160 | Menthol | 0.43  (0.12) | 0.17  (0.02) | 0.14  (0.05) | 0.23  (0.03) | - | 0.06  (0.03) | - | 0.8  (0.14) | 1.38  (0.22) | 0.25  (0.05) | tr. | - | - | 0.23  (0.23) |
| 3 | 12.392 | 1251 | Benzaldehyde  propylene  glycol  acetal | - | - | - | 0.08  (0.05) | - | - | - | 0.11  (0.03) | - | tr. | 0.98  (1.69) | - | - | - |
| 4 | 12.967 | 1295 | 2-Heptyl-1,3-dioxane | 0.88  (0.09) | - | 0.16  (0.09) | tr. | - | tr. | - | 0.36  (0.01) | - | - | - | - | - | tr. |
| **Total acids** | | | | **1.49** | **-** | **-** | **0.14** | **-** | **1.83** | **-** | **0.94** | **-** | **-** | **-** | **-** | **-** | **1.67** |
| 5 | 8.708 | 999 | **Caproic**  **acid** | 1.49  (0.35) | - | - | 0.1  (0.03) | - | 1.33  (0.42) | - | 0.76  (0.21) | - | - | - | - | - | 1.67  (1.67) |
| 6 ^a^ | 11.357 | 1175 | Benzoic  acid | - | - | - | tr. | - | 0.51  (0.21) | - | 0.18  (0.05) | - | - | - | - | - | - |
| **Total alcohols** | | | | **1.6** | **0.18** | **2.54** | **7.63** | **23.1** | **16.64** | **40.58** | **2.33** | **0.37** | **5.17** | **7.62** | **7.15** | **tr.** | **2.68** |
| 7 ^a^ | 5.818 | 838 | 3-Hexen-1-ol | 0.1  (0.04) | tr. | - | 0.35  (0.16) | - | 0.46  (0.24) | 0.72  (1.24) | 0.1  (0.04) | - | 1.01  (0.23) | - | 2.03  (0.13) | - | 0.37  (0.13) |
| 8 ^a^ | 5.91 | 849 | Furfuryl  alcohol | - | - | - | - | - | - | - | - | - | - | - | - | - | 1  (0.04) |
| 9 | 6.017 | 853 | ***(E)*-2-Hexenol** | 0.22  (0.05) | tr. | 0.38  (0.15) | 0.06  (0.04) | 22.07  (1.12) | 2.53  (2.13) | - | - | - | 0.62  (0.03) | - | 0.31  (0.31) | - | - |
| 10 | 6.075 | 857 | **1-Hexanol** | 0.65  (0.1) | 0.11  (0.02) | 0.07  (0.06) | 0.11  (0.04) | 0.94  (0.41) | 4.53  (1.89) | 7.53  (2.31) | tr. | - | 0.07  (0.03) | - | tr. | - | - |
| 11 | 8.869 | 1009 | 2-Ethyl-1-hexanol | 0.28  (0.42) | - | 0.12  (0.06) | 0.53  (0.13) | - | 2.14  (0.46) | 0.48  (0.84) | 0.69  (0.17) | - | tr. | - | - | - | 0.59  (0.59) |
| 12 ^a^ | 9.108 | 1025 | **Benzyl**  **alcohol** | 0.11  (0.07) | - | 0.44  (0.29) | 3.7  (0.81) | - | 4.38  (3.35) | - | 0.68  (0.23) | - | 2.53  (0.16) | 7.5  (5.41) | 0.63  (0.01) | - | 0.31  (0.14) |
| 13 | 9.208 | 1031 | Unknown  alcohol | 0.23  (0.06) | - | 1.51  (1.1) | - | 0.09  (0.09) | 1.37  (0.29) | - | 0.16  (0.05) | - | - | - | - | - | 0.14  (0.14) |
| 14 ^a^ | 9.933 | 1078 | **β-Linalool** | - | tr. | - | tr. | - | 0.99  (0.59) | 31.84  (9.63) | tr. | - | 0.88  (0.23) | 0.09  (0.15) | 4.13  (1.11) | - | 0.27  (0.15) |
| 15 ^a^ | 11.426 | 1180 | α-Terpineol | tr. | - | tr. | 2.85  (2.47) | - | 0.18  (0.1) | - | 0.64  (0.23) | 0.11  (0.04) | tr. | tr. | - | - | - |
| 16 | 20.5 | 1789 | Tetradecamethylene  glycol | - | tr. | - | - | - | 0.06  (0.09) | - | tr. | 0.25  (0) | tr. | - | - | tr. | - |
| **Total aldehydes/furans** | | | | **5.18** | **8.86** | **20.93** | **0.56** | **1.06** | **5.96** | **-** | **1.04** | **14.58** | **tr.** | **0.99** | **0.13** | **1.69** | **11.35** |
| 17 ^a^ | 5.394 | 811 | **Furfural** | - | - | - | - | - | - | - | - | - | - | - | - | - | 4.49  (1.63) |
| 18 | 5.44 | 826 | 3-Furaldehyde | - | - | - | - | - | tr. | - | - | - | tr. | - | - | - | 2.35  (1.32) |
| 19 ^a^ | 7.79 | 947 | Benzaldehyde | - | - | - | 0.16  (0.12) | - | 0.94  (0.56) | - | - | - | tr. | 0.99  (0.72) | 0.13  (0.05) | tr. | - |
| 20 | 7.987 | 958 | 5-methylfurfural | - | - | - | - | - | 0.36  (0.13) | - | - | - | - | - | - | - | 1.75  (0.45) |
| 21 | 11.064 | 1155 | 2-Furfuryl-5-methylfuran | - | - | - | - | - | - | - | 0.44  (0.07) | - | - | - | - | - | - |
| 22 | 11.69 | 1199 | 4,7-Dimethylbenzofuran | - | - | - | tr. | - | - | - | tr. | - | - | - | - | - | - |
| 23 | 12.442 | 1255 | p-Anisaldehyde | 0.15  (0.07) | tr. | - | - | - | 0.09  (0.08) | - | tr. | 0.1  (0.1) | tr. | - | - | 1.64  (0.35) | 0.15  (0.03) |
| 24 ^a^ | 12.75 | 1279 | **Cinnamaldehyde** | 4.12  (0.63) | 8.6  (1.69) | - | 0.07  (0.11) | 1.06  (1.06) | 4.57  (3.22) | - | 0.11  (0.04) | 14.46  (3.44) | - | - | - | - | 0.5  (0.42) |
| 25 | 13.45 | 1336 | Piperonal | tr. | - | 1.04  (0.89) | 0.27  (0.07) | - | - | - | tr. | tr. | - | - | - | - | 2.01  (0.98) |
| 26 | 14.18 | 1392 | Vanillin | 0.27  (0.05) | - | 0.44  (0.57) | - | - | - | - | 0.36  (0.22) | - | - | - | - | - | 0.07  (0.07) |
| 27 | 14.782 | 1443 | **Ethyl**  **Vanillin** | 0.61  (0.03) | 0.22  (0.05) | 19.45  (13.31) | - | - | - | - | tr. | - | - | - | - | - | tr. |
| **Total aromatics** | | | | **tr.** | **tr.** | **-** | **0.83** | **-** | **0.34** | **-** | **0.4** | **-** | **-** | **46.53** | **-** | **tr.** | **0.33** |
| 28 | 10.796 | 1136 | 1-Phenylbutadiene | - | - | - | 0.22  (0.04) | - | - | - | - | - | - | - | - | - | - |
| 29 | 14.064 | 1385 | **Diphenyl**  **ether** | - | - | - | - | - | - | - | - | - | - | 46.53  (4.9) | - | - | - |
| 30 | 14.21 | 1397 | Unknown  aromatic | - | - | - | 0.62  (0.12) | - | - | - | 0.39  (0.17) | - | - | - | - | - | - |
| 31 | 17.142 | 1613 | β-Acetonaphthone | tr. | tr. | - | - | - | 0.34  (0.54) | - | tr. | - | - | - | - | tr. | 0.33  (0.33) |
| **Total esters** | | | | **30.09** | **6.25** | **51.57** | **71.47** | **37.66** | **51.68** | **15.57** | **87.97** | **29.75** | **70.26** | **34.65** | **20.77** | **5.52** | **27.82** |
| 32 | 5.558 | 830 | **Ethyl**  **α-methylbutyrate** | 1.62  (0.64) | tr. | 0.38  (0.15) | 0.06  (0.04) | 22.07  (1.12) | 3.53  (3.75) | - | - | tr. | tr. | 0.58  (0.61) | - | - | - |
| 33 | 6.113 | 858 | Isoamyl  acetate | 0.19  (0.03) | - | 0.2  (0.15) | - | 1.66  (1.66) | 3.48  (2.45) | - | - | - | - | - | - | - | 0.27  (0.19) |
| 34 | 6.15 | 860 | **2-Methylbutyl**  **acetate** | 1.03  (0.14) | 0.13  (0.03) | 1.79  (0.58) | tr. | 13.56  (1.16) | 12.88  (6.38) | 8.12  (7.38) | 0.07  (0.03) | - | 0.72  (0.07) | - | - | - | 6.19  (0.54) |
| 35 | 6.527 | 878 | Propyl  isobutyrate | - | - | 0.15  (0.05) | - | - | - | - | - | - | - | - | - | - | - |
| 36 | 8.242 | 973 | n-Butyl  butyrate | 0.06  (0.04) | - | - | 0.06  (0.01) | - | 0.7  (0.2) | - | tr. | - | tr. | - | - | - | - |
| 37 | 8.292 | 976 | Ethyl  caproate | tr. | - | - | - | - | 1.48  (0.52) | - | - | - | tr. | - | - | - | tr. |
| 38 | 8.525 | 989 | n-Hexyl  acetate | 1.04  (0.26) | - | - | 0.11  (0.02) | - | 0.92  (0.25) | - | - | - | - | - | - | - | tr. |
| 39 | 8.575 | 991 | 2-Hexenol  acetate | 0.19  (0.06) | - | 0.06  (0.03) | tr. | - | 0.92  (0.16) | - | - | - | - | - | - | - | - |
| 40 | 9.225 | 1032 | **iso-Amyl**  **iso-butyrate** | 0.16  (0.03) | - | tr. | 0.81  (0.1) | - | 0.39  (0.01) | - | 35.12  (8.23) | - | 0.18  (0.08) | - | - | - | - |
| 41 | 9.248 | 1034 | **2-Methylbutyl**  **butyrate** | tr. | - | - | 0.42  (0.05) | - | 0.19  (0) | - | 18.64  (4.29) | - | 7.96  (6.75) | - | - | - | - |
| 42 | 9.69 | 1062 | Benzyl  formate | - | - | - | 0.29  (0.25) | - | 1.3  (0.85) | - | tr. | - | 0.27  (0.27) | 0.15  (0.22) | - | - | - |
| 43 | 9.967 | 1079 | Amyl  valerate | - | tr. | - | 0.88  (0.01) | - | 1.45  (0.84) | - | 0.08  (0.04) | - | 0.08  (0.01) | - | - | - | tr. |
| 44 | 10.615 | 1123 | Isobutyl  caproate | - | - | 0.24  (0.07) | 1.2  (0.12) | - | tr. | - | 0.18  (0.06) | - | - | - | - | - | - |
| 45 | 10.908 | 1143 | Benzyl  acetate | 0.11  (0.05) | - | tr. | 0.1  (0.07) | - | 4.03  (1.91) | - | tr. | - | tr. | 0.6  (0.35) | - | - | tr. |
| 46 | 11.088 | 1156 | **Ethylacetoacetate**  **propyleneglycol**  **ketal** | 0.57  (0.08) | 0.19  (0.01) | 2.12  (0.3) | 42.01  (1.84) | 0.3  (0.18) | 4.7  (2.33) | 3.31  (3.91) | 7.24  (2.28) | 1.58  (0.07) | 13.55  (1.88) | 0.15  (0.13) | 0.64  (0) | - | 2.19  (1.68) |
| 47 | 11.108 | 1158 | ***(Z)*-3-Hexenyl**  **butyrate** | 0.22  (0.07) | 0.1  (0.01) | 0.41  (0.21) | 0.69  (0.04) | - | 0.32  (0.16) | - | 0.8  (0.08) | 0.85  (0.11) | 20.37  (3.19) | 0.08  (0.15) | - | - | tr. |
| 48 | 11.172 | 1162 | Butyl  caproate | - | - | - | 0.08  (0.01) | - | 0.3  (0.09) | - | tr. | - | tr. | - | - | - | - |
| 49 | 11.242 | 1167 | ***(E)*-2-Hexenyl**  **butyrate** | 0.41  (0.07) | 0.21  (0.03) | 0.17  (0.13) | 1.74  (0.12) | - | 0.47  (0.12) | - | 0.73  (0.12) | 0.08  (0) | 9.19  (0.87) | - | - | - | 0.1  (0.1) |
| 50 | 11.258 | 1169 | α-Phenylethyl  acetate | tr. | - | 0.08  (0.03) | - | - | - | - | tr. | - | - | - | - | - | - |
| 51 ^a^ | 11.414 | 1179 | Methyl  salicylate | - | - | 0.19  (0.05) | 0.1  (0.07) | - | tr. | - | - | - | - | - | - | - | - |
| 52 ^a^ | 11.967 | 1220 | **Linalyl**  **acetate** | 1.05  (0.11) | - | - | tr. | - | 3.58  (1.46) | - | tr. | 16.24  (1.47) | - | - | - | - | 0.18  (0.18) |
| 53 | 11.973 | 1220 | Isoamyl  caproate | 0.07  (0.01) | - | 0.06  (0.02) | tr. | - | 0.15  (0.05) | - | - | - | - | - | - | - | - |
| 54 | 12.025 | 1224 | **Ethyl**  **phenylacetate** | 12.7  (1.19) | - | 0.1  (0.03) | 0.13  (0.06) | - | 1.14  (0.48) | - | 0.59  (0.11) | 7.38  (0.62) | - | 0.53  (0.83) | - | - | 1.84  (1.82) |
| 55 | 12.23 | 1240 | Benzyl  propionate | 0.38  (0.04) | - | 0.12  (0.03) | tr. | - | 0.07  (0.04) | - | tr. | tr. | tr. | - | - | tr. | tr. |
| 56 | 12.56 | 1265 | Menthyl  acetate | 0.06  (0.01) | 0.11  (0.02) | tr. | - | - | 0.41  (0.19) | - | tr. | 2.72  (2.37) | tr. | - | - | 0.14  (0.05) | 0.09  (0.09) |
| 57 | 12.686 | 1274 | *(Z)*-6-Nonenyl  acetate | 0.22  (0.02) | 0.32  (0.05) | 0.08  (0.03) | tr. | - | 0.6  (0.36) | - | tr. | 0.08  (0) | - | - | - | 0.35  (0.02) | 0.48  (0.31) |
| 58 | 12.967 | 1295 | Propyl  methacrylate | - | - | - | - | - | - | 4.14  (0.84) | - | - | - | - | - | - | - |
| 59 | 13.15 | 1310 | Triacetin | 0.3  (0.07) | - | 3.88  (1.47) | - | - | - | - | - | - | - | - | - | - | tr. |
| 60 | 13.307 | 1323 | Benzyl  butanoate | 8  (6.94) | tr. | tr. | 0.1  (0.03) | - | 0.67  (0.45) | - | 13.83  (4.16) | 0.07  (0.02) | 14.12  (1.39) | 10.75  (0.3) | 1.55  (1.55) | tr. | 1.73  (0) |
| 61 | 13.394 | 1330 | Dihydrocarvyl  acetate | tr. | - | - | 0.12  (0.03) | - | - | - | - | - | - | - | - | - | 2.1  (0.36) |
| 62 | 13.57 | 1344 | Unknown  ester | - | tr. | 0.1  (0.06) | tr. | - | - | - | tr. | tr. | tr. | - | - | - | 0.42  (0.31) |
| 63 | 13.622 | 1349 | *(Z)*-β-Hexenyl  Caproate | tr. | 0.36  (0.1) | 0.42  (0.2) | 1.24  (0.27) | - | 0.14  (0.11) | - | tr. | 0.12  (0) | 0.07  (0) | - | - | - | 1.67  (0.27) |
| 64 | 13.65 | 1351 | Hexyl  caproate | tr. | 0.69  (0.18) | 2.33  (2.82) | tr. | - | 0.34  (0.17) | - | 0.1  (0.09) | tr. | tr. | - | 0.09  (0.01) | - | 1.37  (0.79) |
| 65 | 13.67 | 1352 | Cinnamyl  butyrate  isomer | tr. | 0.27  (0.06) | tr. | 0.29  (0.05) | - | 0.37  (0.22) | - | 0.06  (0.03) | tr. | 2.25  (2.25) | - | - | - | 0.22  (0.1) |
| 66 | 13.685 | 1353 | *(E)*-2-Hexenyl  caproate | tr. | 0.49  (0.12) | 0.19  (0.18) | 0.31  (0.07) | - | 0.62  (0.34) | - | - | - | 0.07  (0.06) | - | - | - | - |
| 67 | 13.763 | 1360 | Ethyl  caprate | tr. | - | 0.4  (0.21) | tr. | - | tr. | - | 1.12  (0.36) | - | - | - | - | - | 0.08  (0.08) |
| 68 | 14.15 | 1392 | **Methyl**  **methanthranilate** | 0.51  (0.05) | - | 26.8  (4.5) | 0.73  (0.28) | - | - | - | - | - | - | - | - | - | tr. |
| 69 | 14.63 | 1432 | Isomenthol  acetate | 0.44  (0.02) | - | - | tr. | - | 0.08  (0.14) | - | 0.12  (0.09) | - | tr. | - | - | - | 0.18  (0.03) |
| 70 | 14.75 | 1441 | *(Z)*-3-Hexenyl  heptanoate | 0.07  (0.01) | tr. | 0.53  (0.38) | tr. | - | tr. | - | tr. | tr. | 0.26  (0.07) | tr. | 0.63  (0.07) | - | 2.48  (1.43) |
| 71 | 14.84 | 1449 | Ethyl  cinnamate | tr. | - | 10.65  (3.52) | 19.18  (4.65) | 0.06  (0.06) | 0.13  (0.07) | - | 0.18  (0.3) | tr. | - | - | 0.34  (0) | - | 0.21  (0.21) |
| 72 | 14.939 | 1456 | **α,α-Dimethylphenethyl**  **butyrate** | - | - | - | - | - | - | - | - | 0.09  (0.01) | tr. | - | 17.01  (1.48) | - | - |
| 73 | 15.09 | 1469 | Geraniol  butyrate | - | - | - | 0.09  (0.01) | - | - | - | - | 0.07  (0) | - | - | - | - | 0.17  (0.17) |
| 74 | 15.24 | 1482 | Citronellyl  butyrate | - | - | - | - | - | - | - | - | tr. | - | 0.5  (0.06) | 0.5  (0.2) | - | - |
| 75 | 15.26 | 1483 | Hydrocinnamyl  isobutyrate | - | - | - | 0.14  (0.13) | - | - | - | tr. | tr. | - | - | - | - | 0.14  (0.14) |
| 76 | 15.47 | 1500 | Cinnamyl  butyrate | tr. | - | - | 0.12  (0.02) | - | 0.13  (0.19) | - | tr. | tr. | 0.23  (0.04) | 1.99  (0.25) | - | - | 0.28  (0.2) |
| 77 | 15.68 | 1515 | Benzyl  hexanoate | - | 0.07  (0) | - | 0.18  (0.03) | - | 0.37  (0.41) | - | - | tr. | tr. | 7.78  (0.7) | - | - | 0.11  (0.11) |
| 78 | 15.7 | 1517 | Geranyl  isobutyrate | tr. | tr. | - | tr. | - | tr. | - | 0.08  (0.02) | tr. | - | 0.58  (1) | - | - | 0.31  (0.27) |
| 79 | 16.258 | 1555 | **Cinnamyl**  **isobutyrate** | - | - | - | tr. | - | tr. | - | 8.62  (4.27) | - | - | - | - | - | tr. |
| 80 | 17.11 | 1611 | Benzyl  n-heptanoate | - | tr. | - | - | - | - | - | - | 0.08  (0.06) | tr. | 10.91  (1.78) | - | tr. | - |
| 81 | 17.25 | 1618 | **Hedione**  **(Methyl**  **dihydrojasmonate)** | 0.47  (0.17) | 3.17  (0.4) | - | - | - | 0.13  (0.19) | - | - | tr. | 0.65  (0.38) | - | - | 0.16  (0.15) | 4.76  (2.56) |
| 82 | 17.358 | 1619 | α-Amylcinnamaldehyde | - | - | - | - | - | 5.44  (6.76) | - | - | - | - | - | - | 4.8  (4.61) | - |
| **Total ketones** | | | | **1.52** | **0.13** | **5.45** | **1.51** | **15.22** | **17.5** | **13.19** | **0.45** | **51.42** | **0.65** | **tr.** | **0.49** | **0.07** | **17.19** |
| 83 ^a^ | 6.125 | 859 | **2,3-Butanedione** | 1.03  (0.14) | 0.1  (0.02) | 1.66  (0.54) | tr. | 13.56  (1.16) | 12.88  (6.38) | 11.47  (1.31) | 0.07  (0.03) | - | 0.57  (0.02) | - | 0.49  (0.03) | - | 6.19  (0.54) |
| 84 | 6.158 | 861 | Butenone | 0.19  (0.03) | - | 0.2  (0.15) | - | 1.66  (1.66) | 3.48  (2.45) | - | - | - | - | - | - | - | 0.27  (0.19) |
| 85 | 8.108 | 965 | Sulcatone (6-Methyl-5-hepten-2-one) | - | - | 0.12  (0.08) | - | - | 0.45  (0.04) | 1.71  (2.97) | - | - | - | - | - | - | 0.9  (0.21) |
| 86 | 9.925 | 1077 | Furaneol | - | - | - | - | - | - | - | - | - | tr. | - | - | - | - |
| 87 | 10.966 | 1147 | Pyranone | - | - | - | 0.84  (0.06) | - | - | - | 0.06  (0.03) | - | - | - | - | - | - |
| 88 | 11.943 | 1218 | 5-Acetoxymethyl-2-furaldehyde | tr. | - | 0.71  (0.11) | - | - | - | - | - | - | - | - | - | - | - |
| 89 | 12.017 | 1223 | Pulegone | 0.09  (0) | - | 0.51  (0.17) | - | - | 0.53  (0.19) | - | tr. | 2.02  (0.19) | - | - | - | - | 0.07  (0.01) |
| 90 ^a^ | 12.125 | 1231 | **Carvone** | - | - | - | - | - | - | - | - | 48.01  (4.29) | - | - | - | - | - |
| 91 | 12.31 | 1246 | 2-Hydroxy-3-isopropyl-6-methyl-2-cyclohexen-1-one | - | - | 0.68  (0.12) | - | - | 0.06  (0.04) | - | - | - | - | - | - | - | - |
| 92 | 12.76 | 1280 | Diosphenol | 0.09  (0.01) | - | 1.19  (0.19) | - | - | - | - | 0.12  (0.03) | - | - | - | - | - | 0.13  (0.06) |
| 93 | 13.4 | 1331 | ***(±)*-Solanone** | tr. | - | tr. | 0.13  (0.02) | - | tr. | - | tr. | 0.22  (0) | tr. | - | - | - | 7.19  (0.42) |
| 94 | 14.26 | 1347 | γ-Ionone | - | tr. | 0.26  (0.08) | 0.14  (0.02) | - | - | - | 0.08  (0.06) | 0.42  (0.39) | - | - | - | 0.07  (0.04) | 0.69  (0.38) |
| 95 | 14.45 | 1415 | Geranyl  acetone | tr. | - | 0.06  (0.05) | 0.35  (0.11) | - | 0.09  (0.13) | - | tr. | 0.75  (0.14) | tr. | tr. | - | - | 1.75  (1.15) |
| **Total lactones** | | | | **-** | **1.48** | **-** | **-** | **-** | **-** | **-** | **-** | **0.08** | **20.84** | **8.22** | **67.3** | **tr.** | **-** |
| 96 | 7.065 | 901 | Butyrolactone | - | - | - | - | - | - | - | - | - | - | - | - | - | - |
| 97 | 14.814 | 1447 | **γ-Decalactone** | - | 1.45  (0.14) | - | - | - | - | - | - | tr. | 20.58  (5.63) | 7.59  (3.33) | 50.82  (0.12) | - | - |
| 98 | 16.127 | 1545 | **γ-Undecalactone** | - | tr. | - | - | - | - | - | - | tr. | 0.27  (0.1) | 0.63  (0.31) | 16.48  (2.36) | tr. | - |
| **Total monoterpene hydrocarbons** | | | | **0.13** | **-** | **2.23** | **0.16** | **0.32** | **1.37** | **-** | **1.49** | **0.19** | **-** | **0.79** | **-** | **-** | **0.42** |
| 99 ^a^ | 8.122 | 966 | β-Myrcene | tr. | - | 0.08  (0.1) | - | - | 0.17  (0.04) | - | - | - | - | - | - | - | - |
| 100 ^a^ | 8.825 | 1007 | Limonene | 0.06  (0.01) | - | 1.19  (0.65) | 0.12  (0.01) | 0.32  (0.32) | 0.95  (0.59) | - | 1.3  (0.32) | tr. | - | 0.79  (1.23) | - | - | 0.07  (0.07) |
| 101 ^a^ | 8.891 | 1011 | *(E)*-β-Ocimene | tr. | - | 0.83  (0.55) | tr. | - | 0.06  (0.03) | - | - | 0.15  (0.1) | - | - | - | - | 0.35  (0.35) |
| 102 | 9.065 | 1022 | *(Z)*-β-Ocimene | - | - | 0.11  (0.07) | - | - | - | - | 0.09  (0.08) | - | - | - | - | - | - |
| 103 | 9.725 | 1064 | Isoterpinolene | - | - | - | - | - | 0.19  (0.11) | - | 0.09  (0.08) | - | - | - | - | - | - |
| **Total nitrogenous compounds/alkaloids** | | | | **0.26** | **0.59** | **0.39** | **0.17** | **22.07** | **2.72** | **0.21** | **-** | **tr.** | **1.05** | **-** | **-** | **-** | **27.69** |
| 104 | 6.147 | 860 | **Pyrrolidine** | 0.22  (0.05) | tr. | 0.38  (0.15) | 0.06  (0.04) | 22.07  (1.12) | 2.6  (2.07) | - | - | - | 0.95  (0.15) | - | - | - | - |
| 105 | 7.096 | 907 | Nitrosoazetidine | tr. | - | - | - | - | 0.12  (0.05) | - | - | - | - | - | - | - | 0.96  (0.42) |
| 106 ^a^ | 13.585 | 1345 | **Nicotine** | tr. | 0.58  (0.15) | tr. | 0.11  (0.03) | - | - | 0.21  (0.37) | - | tr. | 0.1  (0) | - | - | - | 26.73  (10.45) |
| **Total oxygenated monoterpenes** | | | | **57.78** | **82.31** | **2.61** | **0.81** | **0.58** | **0.5** | **30.45** | **2.57** | **0.27** | **1.65** | **0.16** | **4.17** | **92.68** | **10.1** |
| 107 ^a^ | 8.9 | 1012 | Cineole | - | - | - | tr. | - | - | - | - | tr. | - | - | - | - | - |
| 108 | 9.925 | 1077 | Diepoxy-p-Menthane | - | tr. | - | - | - | - | 30.45  (6.23) | - | - | 1.56  (0.47) | 0.16  (0.15) | 4.17  (0.15) | - | - |
| 109 | 10.817 | 1146 | Isomenthone | - | - | 2.33  (1.01) | 0.19  (0.03) | - | - | - | - | 0.19  (0.02) | - | - | - | - | - |
| 110 ^a^ | 12.675 | 1273 | ***(E)-*Anethole** | 57.78  (5.56) | 82.29  (1.95) | 0.28  (0.2) | 0.6  (0.27) | 0.58  (0.58) | 0.5  (0.63) | - | 2.57  (0.4) | tr. | 0.1  (0.08) | - | - | 92.68  (5.23) | 10.1  (9.77) |
| **Total phenols** | | | | **0.59** | **-** | **13.98** | **0.89** | **-** | **1.34** | **-** | **-** | **tr.** | **-** | **-** | **-** | **-** | **0.5** |
| 111 | 8.433 | 983 | Phenol | 0.59  (0.04) | - | 0.73  (0.62) | 0.1  (0.01) | - | 1.34  (0.22) | - | - | - | - | - | - | - | 0.32  (0.32) |
| 112 ^a^ | 13.45 | 1334 | **Eugenol** | - | - | 13.25  (11.42) | 0.79  (0.23) | - | - | - | - | tr. | - | - | - | - | 0.18  (0.18) |
| **Total sesquiterpene hydrocarbons** | | | | **-** | **tr.** | **-** | **-** | **-** | **-** | **-** | **-** | **1.9** | **-** | **-** | **-** | **-** | **-** |
| 113 | 13.767 | 1361 | *(-)*-β-Bourbonene | - | - | - | - | - | - | - | - | 0.58  (0) | - | - | - | - | - |
| 114 ^a^ | 14.225 | 1398 | Caryophyllene | - | tr. | - | - | - | - | - | - | 1.32  (0.05) | - | - | - | - | - |

^a^ Confirmed by comparing to authentic standard analysed under same conditions.

^b^ tr. Traces, present but ≤ 0.05.

EG, Egyptian hookah tobacco; EM, United Arab Emirates hookah tobacco; Rt, Retention time; KI, Kovats index.

The total percentile levels for each class and major volatiles are bolded.

**Supplementary Table S2** Relative percentage of volatile components detected in the volatile blend of superheated (at 190°C) guava, watermelon, peach, mango and melon hookah tobacco products and cigarette tobacco product as analysed using SPME-GC-MS (n = 3).

| **Peak Number** | **Rt** | **KI** | **Compound Name** | **GUAVA** | **MELON** | **WATERMELON** | **PEACH** | **MANGO** | **ROTHMANS CIGARETTE** |
| --- | --- | --- | --- | --- | --- | --- | --- | --- | --- |
|  |  |  |  | **Average (Standard Deviation)** | | | | |  |
| **Total alcohols** | | | | **1.03** | **8.61** | **1.67** | **4.92** | **7.14** | **tr.** |
| 1 | 6.378 | 871 | Ethylene glycol, monoacetate | - | - | - | 1.6 (1.43) | tr. ^b^ | - |
| 2 | 9.13 | 1026 | Benzyl alcohol | 1 (0.1) | 0.95 (0.77) | 0.43 (0.19) | 1.58 (0.59) | 5.57 (0.88) | - |
| 3 | 10.458 | 1112 | Monoacetyl glycerine | - | - | - | 0.88 (0.84) | 0.95 (0.36) | - |
| 4 | 20.5 | 1789 | Tetradecamethylene glycol | tr. | 7.66 (1.59) | 1.24 (0.3) | 0.85 (0.78) | 0.59 (0.09) | tr. |
| 5 | 5.91 | 849 | Furfuryl alcohol | tr. | - | 0.21 (0.05) | 0.67 (0.35) | - | - |
| **Total aldehydes** | | | | **0.27** | **0.43** | **2.14** | **3.17** | **4.91** | **tr.** |
| 6 ^a^ | 7.833 | 949 | Benzaldehyde | 0.17 (0.04) | 0.42 (0.52) | 0.34 (0.07) | 0.73 (0.3) | 3.89 (0.29) | - |
| 7 | 7.834 | 949 | 5-Methylfurfural | 0.1 (0.01) | tr. | 1.8 (0.3) | 2.43 (1.62) | 1.02 (0.06) | - |
| 8 | 9.225 | 1032 | iso-Amyl iso-butyrate | 0.13 (0.05) | 0.68 (0.45) | 0.54 (0.18) | 0.18 (0.04) | 0.19 (0.07) | - |
| 9 | 11.003 | 1150 | Ethylacetoacetate propyleneglycol ketal | - | - | - | 1.78 (0.22) | 0.67 (0.51) | - |
| 10 ^a^ | 12.56 | 1265 | Cinnamaldehyde | 0.5 (0.19) | - | 0.6 (0.24) | 0.06 (0.04) | 0.4 (0.25) | - |
| **Total esters** | | | | **33.79** | **36.7** | **13.32** | **5.73** | **13.87** | **0.11** |
| 11 | 12.686 | 1274 | *(Z)*-6-Nonenyl acetate | 0.75 (0.11) | 0.61 (0.64) | 0.76 (0.05) | 0.41 (0.65) | 0.88 (0.29) | - |
| 12 | 13.307 | 1323 | Benzyl butanoate | 1.19 (0.04) | 0.29 (0.16) | 1.25 (0.13) | 0.15 (0.06) | 1.52 (0.4) | tr. |
| 13 | 13.316 | 1323 | Ethyl dihydrocinnamate | 1.26 (0.11) | 0.06 (0.03) | 0.29 (0.03) | tr. | tr. | - |
| 14 | 13.606 | 1347 | 2,6-Dimethyl-2,6-octadien-8-yl acetate | 0.3 (0.04) | 0.9 (1) | 0.85 (0.14) | 2.58 (2.92) | 0.14 (0.07) | tr. |
| 15 | 13.642 | 1350 | Hexyl caproate | 0.42 (0) | 0.87 (0.37) | 0.74 (0.07) | 0.09 (0.03) | 0.13 (0.05) | tr. |
| 16 | 13.685 | 1353 | *(E)*-2-Hexenyl caproate | 0.13 (0.02) | 1.15 (0.52) | 0.23 (0.04) | tr. | tr. | tr. |
| 17 | 14.84 | 1449 | Ethyl cinnamate | 27.84 (11.14) | 0.33 (0.39) | 0.93 (0.12) | 0.11 (0.09) | 0.56 (0.36) | tr. |
| 18 | 15.47 | 1500 | Cinnamyl butyrate | 0.2 (0.02) | 0.51 (0.61) | 0.47 (0.04) | tr. | 0.07 (0.05) | - |
| 19 | 15.708 | 1517 | Benzyl n-hexanoate | 0.52 (0.16) | 0.18 (0.21) | 0.52 (0.04) | tr. | 2.92 (0.05) | tr. |
| 20 | 16.258 | 1555 | Cinnamyl isobutyrate | 0.26 (0.13) | 0.08 (0.11) | 4.8 (0.44) | 0.09 (0.13) | 0.36 (0.07) | tr. |
| 21 | 17.1 | 1610 | Benzyl n-heptanoate | - | - | - | 0.08 (0.06) | 5.63 (0.8) | - |
| 22 | 17.358 | 1619 | α-Amylcinnamaldehyde | 0.22 (0.13) | 20.81 (10.21) | 0.45 (0.09) | 0.06 (0.09) | 0.09 (0.02) | - |
| 23 | 17.507 | 1631 | *(Z)*-6-Nonenyl acetate | tr. | 0.55 (0.54) | 0.71 (0.42) | tr. | 0.23 (0.02) | tr. |
| 24 | 17.746 | 1619 | α-Amylcinnamaldehyde isomer | tr. | 9.64 (5.2) | 0.18 (0.06) | - | - | - |
| **Total aromatics** | | | | **10.63** | **2.01** | **19.4** | **4.38** | **12.18** | **0.15** |
| 25 | 5.775 | 842 | Ethylbenzene | 0.11 (0.02) | - | 0.13 (0.04) | 0.13 (0.11) | 0.75 (0.36) | - |
| 26 | 6.4 | 872 | **m-Xylene** | tr. | - | 0.13 (0.03) | tr. | 0.51 (0.21) | - |
| 27 | 7.65 | 939 | Cumene | 0.08 (0) | - | 0.32 (0.06) | 0.69 (0.29) | 0.55 (0.22) | - |
| 28 ^a^ | 8.739 | 1001 | p-Cymene | tr. | tr. | 0.58 (0.26) | 0.15 (0.15) | 0.6 (0.17) | - |
| 29 | 8.828 | 1008 | *(Z)*-β-Methylstyrene | tr. | - | 0.53 (0.11) | tr. | 0.21 (0.08) | - |
| 30 | 9.25 | 1034 | n-Butylbenzene | 0.14 (0.01) | 0.06 (0.07) | 0.29 (0.03) | 0.64 (0.13) | 2.71 (0.54) | - |
| 31 | 9.747 | 1065 | p-Ethylstyrene | 0.07 (0.03) | - | 0.26 (0.04) | 0.34 (0.46) | 0.42 (0.01) | - |
| 32 | 10.33 | 1103 | 2-Methylallyl-benzene | tr. | - | 0.19 (0.07) | 0.61 (0.92) | 0.2 (0.02) | - |
| 33 | 10.567 | 1120 | 1-Methylindan | 0.07 (0.02) | 0.1 (0.13) | 0.2 (0.02) | 0.12 (0.1) | 0.37 (0.02) | - |
| 34 | 10.72 | 1132 | 2-Methylindene | 0.21 (0.07) | - | 0.56 (0.09) | 0.06 (0.05) | 0.37 (0.01) | tr. |
| 35 | 10.73 | 1131 | **3-Methylindene** | 0.14 (0.03) | - | 0.17 (0.03) | tr. | 0.14 (0.05) | - |
| 36 | 11.31 | 1172 | Naphthalene | 0.29 (0.12) | 0.69 (0.84) | 0.51 (0.1) | 0.1 (0.06) | 0.3 (0.03) | - |
| 37 | 12.343 | 1251 | **1,2-Dihydro-4-methyl-naphthalene** | 1.07 (0.3) | tr. | 2.32 (0.36) | 0.11 (0.09) | 0.69 (0.1) | - |
| 38 | 12.717 | 1279 | 1,1,3-Trimethyl-indan | 0.53 (0.14) | tr. | 1.26 (0.2) | 0.35 (0.44) | 0.66 (0.11) | tr. |
| 39 | 12.8 | 1283 | 1-Methylnaphthalene | 1.34 (0.43) | 0.11 (0.13) | 1.51 (0.16) | 0.17 (0.12) | 0.46 (0.07) | tr. |
| 40 | 12.944 | 1293 | 1-Methylindan-2-one | 0.8 (0.07) | 0.14 (0.15) | 0.62 (0.08) | 0.12 (0.1) | 0.59 (0.23) | tr. |
| 41 | 13 | 1299 | 1-Methylnaphthalene isomer | 0.71 (0.08) | - | 0.64 (0.08) | 0.1 (0.1) | 0.54 (0.14) | - |
| 42 | 14.163 | 1393 | **2,7-Dimethylnaphthalene** | 0.7 (0.05) | 0.1 (0.12) | 1.12 (0.11) | 0.1 (0.07) | 0.28 (0.07) | tr. |
| 43 | 14.375 | 1409 | **2,6-Dimethylnaphthalene** | 1.71 (0.09) | tr. | 3.14 (0.23) | 0.23 (0.17) | 0.53 (0.18) | tr. |
| 44 | 14.568 | 1436 | **1,4-Dimethylnaphthalene** | 0.63 (0.04) | - | 1.18 (0.08) | tr. | 0.33 (0.11) | tr. |
| 45 | 15.06 | 1466 | p-Methylbiphenyl | 0.24 (0.02) | - | 0.33 (0.03) | tr. | 0.08 (0.03) | - |
| 46 | 15.153 | 1474 | Acenaphthene | 0.36 (0.05) | - | 0.45 (0.08) | tr. | 0.1 (0.03) | tr. |
| 47 | 15.25 | 1483 | 2,5,8-Trimethyl-1,2,3,4-tetrahydro-1-naphthol | 0.59 (0.14) | 0.66 (0.77) | 0.73 (0.12) | tr. | 0.18 (0.02) | tr. |
| 48 | 15.57 | 1507 | 2,3,5-Trimethyl-naphthalene | 0.73 (0.25) | tr. | 2.22 (0.25) | 0.19 (0.16) | 0.6 (0.2) | tr. |
| **Total furans** | | | | **2.49** | **0.44** | **8.9** | **1.44** | **3.01** | **0.11** |
| 49 | 8.042 | 961 | Unknown furan | 0.14 (0.02) | 0.11 (0.04) | 0.47 (0.13) | 0.52 (0.55) | 0.61 (0.06) | tr. |
| 50 | 10.14 | 1091 | 2-Methylbenzofuran | 0.21 (0.1) | tr. | 1.04 (0.22) | 0.19 (0.16) | 0.73 (0.06) | - |
| 51 | 11.63 | 1195 | 4,7-Dimethylbenzofuran | 1.26 (0.42) | - | 4.04 (0.61) | 0.29 (0.22) | 0.74 (0.01) | tr. |
| 52 | 11.726 | 1201 | 2-Ethylbenzofuran | 0.29 (0.09) | - | 0.8 (0.03) | 0.06 (0.05) | 0.29 (0.07) | - |
| 53 | 12.285 | 1243 | 2,2'-methylenebis[5-methyl- Furan] | 0.14 (0.02) | - | 1.8 (0.45) | 0.23 (0.13) | 0.42 (0.03) | - |
| 54 | 15.6 | 1509 | Dibenzofuran | 0.44 (0.05) | 0.29 (0.32) | 0.74 (0.07) | 0.16 (0.1) | 0.23 (0.08) | - |
| **Total hydrocarbons** | | | | **1.75** | **1.56** | **6.95** | **19.75** | **4.17** | **0.13** |
| 55 ^a^ | 14.9 | 1453 | Pentadecane | 1.4 (0.62) | 0.4 (0.44) | 3.82 (1.14) | 0.39 (0.24) | 1.38 (0.65) | 0.1 (0) |
| 56 ^a^ | 16.183 | 1553 | Tridecane | 0.15 (0.06) | 0.48 (0.46) | 1.92 (0.51) | 18.14 (3.19) | 1.6 (0.21) | tr. |
| 57 ^a^ | 17.78 | 1650 | Hexadecane | 0.17 (0.11) | 0.61 (0.34) | 1 (0.27) | 0.94 (0.45) | 0.98 (0.52) | tr. |
| 58 | 19.908 | 1758 | Unknown hydrocarbon | tr. | 0.07 (0.02) | 0.2 (0.06) | 0.27 (0.16) | 0.21 (0.1) | - |
| **Total ketones** | | | | **3.42** | **2.66** | **6.37** | **1.88** | **6.29** | **tr.** |
| 59 | 5.463 | 827 | 2-Methylcyclopentanone | - | - | - | 0.23 (0.33) | 0.38 (0.03) | - |
| 60 | 6.76 | 890 | .2-Methyl-2-cyclopentenone isomer | - | - | - | 0.36 (0.31) | 0.89 (0.16) | - |
| 61 | 7.35 | 925 | 4,4-Dimethyl-2-cyclopenten-1-one | tr. | - | 0.2 (0.03) | 0.07 (0.07) | 1.45 (0.25) | - |
| 62 | 9.008 | 1019 | 3-methyl-1,2-cyclopentanedione | 0.24 (0.07) | tr. | 0.61 (0.15) | 0.51 (0.54) | 0.56 (0.08) | - |
| 63 | 9.39 | 1043 | 3,4-Dimethyl-2-hydroxy-2-cyclopentenone | 0.24 (0.03) | - | 0.46 (0.08) | 0.15 (0.14) | 0.43 (0.19) | - |
| 64 | 9.4 | 1045 | 2,4-Dimethyl-1,3-cyclopentanedione | 1.19 (0.25) | 2.26 (2.47) | 0.33 (0.06) | 0.25 (0.2) | 1.32 (0.23) | - |
| 65 | 10.373 | 1114 | 2-Hydroxy-3-ethyl-2-cyclopenten-1-one | 0.2 (0.04) | 0.13 (0.17) | 0.2 (0.05) | 0.09 (0.09) | 0.49 (0.11) | - |
| 66 | 12.66 | 1275 | 1-Indanone | 0.55 (0.1) | 0.1 (0.04) | 0.52 (0.05) | 0.1 (0.07) | 0.29 (0.08) | tr. |
| 67 | 14.29 | 1403 | 3,3-Dimethyl-1-indanone | 0.72 (0) | - | 1.35 (0.36) | tr. | 0.24 (0.08) | tr. |
| 68 | 16.3 | 1558 | Megastigmatrienone | 0.16 (0.06) | - | 1.01 (0.27) | tr. | 0.14 (0.06) | tr. |
| 69 | 17.2 | 1616 | Hedione | 0.07 (0.02) | 0.15 (0.12) | 1.7 (0.45) | 0.06 (0.04) | 0.09 (0.05) | - |
| **Total lactones** | | | | **7.53** | **3.37** | **7.67** | **32.01** | **16.19** | **tr.** |
| 70 | 14.789 | 1444 | **γ-Decalactone** | - | - | - | 11.54 (10.53) | 4.95 (0.12) | tr. |
| 71 | 14.85 | 1449 | **γ-Decalactone isomer** | 4.11 (1.88) | 0.71 (0.82) | 1.3 (0.37) | 18.59 (21) | 4.95 (0.12) | - |
| 72 | 16.134 | 1547 | **γ-Undecalactone** | - | - | - | 18.14 (3.19) | 1.6 (0.21) | - |
| **Total nitrogenous compounds/alkaloids** | | | | **1.13** | **3.29** | **2.42** | **0.38** | **1.51** | **98.79** |
| 73 | 8.258 | 973 | N,N'-Dimethylthiourea | 0.07 (0.02) | 3.28 (2.34) | 0.24 (0.04) | 0.07 (0.12) | 0.67 (0.27) | - |
| 74 | 11.67 | 1197 | 2,3-diethenyl-5-methylpyrazine | 1.05 (0.37) | tr. | 2.18 (0.31) | 0.31 (0.24) | 0.85 (0.01) | - |
| 111 | 12.935 | 1295 | Indole | - | - | - | - | - | 0.06 (0.02) |
| 112 | 13.467 | 1344 | Nicotine | - | - | - | - | - | 98.73 (0.35) |
| **Total phenols** | | | | **36.42** | **42.66** | **25.78** | **8.12** | **27.24** | **0.44** |
| 75 | 8.408 | 974 | **Phenol** | 0.53 (0.09) | 0.08 (0.1) | 1.16 (0.97) | 2.15 (1.91) | tr. | tr. |
| 76 | 9.467 | 1048 | **o-Cresol** | 1.72 (0.49) | 0.7 (0.85) | 1.34 (1.15) | 1.59 (1.41) | 10.35 (7.17) | tr. |
| 77 | 9.843 | 1071 | **p-Cresol** | 1.58 (0.66) | 9.45 (3.56) | 0.93 (0.33) | tr. | 3.15 (0.53) | tr. |
| 78 | 9.858 | 1073 | **m-Cresol** | 0.6 (0.19) | 8.84 (3.43) | 0.47 (0.23) | 0.13 (0.02) | 0.34 (0.05) | - |
| 79 | 9.867 | 1075 | **o-Guaiacol** | 1.57 (0.82) | 0.35 (0.36) | 0.94 (0.31) | 0.55 (0.41) | 0.87 (0.25) | tr. |
| 80 | 10.067 | 1086 | **Cresol** | 0.31 (0.05) | tr. | 0.12 (0.06) | 0.11 (0.07) | 0.27 (0.05) | tr. |
| 81 | 10.187 | 1093 | o-Xylenol | 0.28 (0.07) | 10.15 (6.69) | 1.25 (0.37) | 0.18 (0.13) | 0.38 (0.02) | tr. |
| 82 | 10.65 | 1125 | Ethylphenol | 0.79 (0.3) | 1.29 (0.51) | 0.68 (0.12) | 0.21 (0.19) | 0.68 (0.01) | - |
| 83 | 10.83 | 1138 | p-Xylenol | 2.93 (1.15) | 0.15 (0.18) | 1.85 (0.35) | 0.49 (0.46) | 1.55 (0.27) | tr. |
| 84 | 10.921 | 1145 | **3,5-Xylenol** | 2.35 (0.9) | 0.89 (0.97) | 1.48 (0.23) | 0.6 (0.49) | 1.94 (0.37) | tr. |
| 85 | 11.207 | 1165 | **p-Ethylphenol** | 1.72 (0.63) | 0.13 (0.07) | 1.6 (0.41) | 0.14 (0.13) | 0.53 (0.09) | tr. |
| 86 | 11.22 | 1166 | o-Ethylphenol isomer | 2.26 (1.11) | 0.7 (0.85) | 1.81 (0.5) | 0.52 (0.37) | 1.14 (0.31) | 0.06 (0.03) |
| 87 | 11.54 | 1189 | Mesityl alcohol | 0.87 (0.29) | tr. | 0.54 (0.2) | 0.16 (0.14) | 0.6 (0.15) | - |
| 88 | 11.8 | 1207 | o-Propylphenol | 0.63 (0.25) | 5.33 (6.93) | 0.61 (0.12) | 0.1 (0.07) | 0.4 (0.14) | tr. |
| 89 | 11.93 | 1217 | 2,3,6-Trimethylphenol | 0.98 (0.31) | - | 0.65 (0.14) | 0.15 (0.12) | 0.55 (0.11) | tr. |
| 90 | 12.01 | 1223 | **Ethyl-m-cresol** | 1.75 (0.7) | tr. | 1.01 (0.14) | 0.17 (0.13) | 0.79 (0.08) | tr. |
| 91 | 12.35 | 1248 | 2-Methyl-1,2-dihydronaphthalene | 2.34 (0.44) | tr. | 0.79 (0.21) | 0.1 (0.03) | 0.25 (0.03) | tr. |
| 92 | 12.43 | 1255 | 3,4,5-Hemimellitenol | 2.65 (0.57) | - | 1.24 (0.23) | 0.07 (0.04) | 0.28 (0.05) | tr. |
| 93 | 12.442 | 1257 | p-Ethylguaiacol | 2.29 (0.55) | tr. | 1.45 (0.18) | 0.11 (0.09) | 0.43 (0.18) | tr. |
| 94 | 12.486 | 1259 | Pseudocumenol | 0.4 (0.08) | 3.23 (5.57) | 0.39 (0.07) | tr. | 0.31 (0.09) | - |
| 95 | 12.592 | 1267 | p-Cymen-7-ol | 0.21 (0.07) | - | 0.38 (0.03) | tr. | 0.11 (0.03) | - |
| 96 | 12.615 | 1269 | o-Acetylphenol | 2.17 (0.4) | 0.53 (0.82) | 0.98 (0.32) | 0.16 (0.2) | 1.11 (0.24) | tr. |
| 97 | 13.079 | 1304 | 3,5-Diethylphenol | 1.32 (0.26) | tr. | 1.39 (0.27) | tr. | 0.11 (0.01) | tr. |
| 98 | 13.15 | 1311 | Paroxypropione | 0.81 (0.08) | 0.63 (0.79) | 0.79 (0.15) | 0.11 (0.07) | 0.46 (0.23) | tr. |
| 99 | 13.45 | 1334 | **Eugenol** ^a^ | 1.46 (0.1) | - | 0.48 (0.04) | tr. | 0.21 (0.07) | tr. |
| 100 | 13.578 | 1345 | Tetramethyl-phenol | 1.27 (0.03) | - | 0.92 (0.02) | tr. | 0.29 (0.08) | tr. |
| 101 ^a^ | 14.6 | 1429 | *(Z)*-Isoeugenol | 0.63 (0.01) | - | 0.55 (0.06) | 0.07 (0.06) | 0.14 (0.05) | tr. |
| **Total phenolic ethers** | | | | **4.62** | **0.21** | **6.82** | **0.79** | **5.71** | **tr.** |
| 102 | 11.525 | 1187 | p-Propargyloxytoluene | 0.65 (0.22) | - | 1.81 (0.25) | 0.25 (0.21) | 0.8 (0.05) | - |
| 103 | 11.342 | 1174 | **4-methoxy-3-methyl-phenol** | 2.07 (0.87) | 0.07 (0.09) | 1.39 (0.25) | 0.13 (0.06) | 0.23 (0.09) | tr. |
| 104 | 14.04 | 1383 | **Diphenyl ether** | - | - | - | - | 3.86 (0.71) | - |
| 105 | 15.844 | 1526 | 2,7-dimethoxy-naphthalene | 0.39 (0.15) | 0.06 (0.08) | 2.46 (0.42) | 0.36 (0.24) | 0.59 (0.11) | - |
| 106 | 16.34 | 1561 | p-1-Cyclohexen-1-yl-anisole | 0.34 (0) | tr. | 0.59 (0.11) | tr. | 0.1 (0) | - |
| 107 | 13.54 | 1342 | p-Propylguaiacol | 1.16 (0.24) | 0.06 (0.02) | 0.58 (0.02) | tr. | 0.14 (0.08) | - |
| **Total terpenes** | | | | **0.33** | **0.71** | **4.72** | **0.5** | **2.47** | **0.21** |
| 108 ^a^ | 8.8 | 1006 | Limonene | 0.07 (0) | 0.38 (0.27) | 1.22 (0.31) | 0.13 (0.13) | 1.36 (0.46) | tr. |
| 109 | 14.48 | 1419 | 5,9,13-Trimethyl-tetradecatrienal | 0.26 (0.07) | 0.07 (0.06) | 3.35 (0.97) | 0.37 (0.24) | 1.11 (0.44) | 0.16 (0.01) |
| 110 | 21.692 | 1851 | *(Z,E)*-α-Farnesene | - | 0.27 (0.34) | 0.15 (0.1) | tr. | - | - |

^a^ Confirmed by comparing to authentic standard analysed under same conditions.

^b^ tr. Traces, present but ≤ 0.05.

Rt, Retention time; KI, Kovats index.

The total percentile levels for each class and major volatiles are bolded.
